# The evolution of paralogous enzymes MAT and MATX within the Euglenida and beyond

**DOI:** 10.1186/1471-2148-14-25

**Published:** 2014-02-11

**Authors:** Jana Szabová, Naoji Yubuki, Brian S Leander, Richard E Triemer, Vladimír Hampl

**Affiliations:** 1 Department of Parasitology, Charles University in Prague, Faculty of Science, Vinicna 7, Prague 2 128 44, Czech Republic; 2 Biotechnology and Biomedicine Center of the Academy of Sciences and Charles University in Vestec, Prague, Czech Republic; 3 Departments of Botany and Zoology, Canadian Institute for Advanced Research, Program in Integrated Microbial Biodiversity, University of British Columbia, Vancouver, British Columbia V6T 1Z4, Canada; 4 Department of Plant Biology, Michigan State University, East Lansing, Michigan 48824, USA

**Keywords:** Methionine adenosyltransferase, Horizontal gene transfer, Deep paralogy, Gene evolution, Euglenozoa

## Abstract

**Background:**

Methionine adenosyltransferase (MAT) is a ubiquitous essential enzyme that, in eukaryotes, occurs in two relatively divergent paralogues: MAT and MATX. MATX has a punctate distribution across the tree of eukaryotes and, except for a few cases, is mutually exclusive with MAT. This phylogenetic pattern could have arisen by either differential loss of old paralogues or the spread of one of these paralogues by horizontal gene transfer. Our aim was to map the distribution of MAT/MATX genes within the Euglenida in order to more comprehensively characterize the evolutionary history of MATX.

**Results:**

We generated 26 new sequences from 23 different lineages of euglenids and one prasinophyte alga *Pyramimonas parkeae*. MATX was present only in photoautotrophic euglenids. The mixotroph *Rapaza viridis* and the prasinophyte alga *Pyramimonas parkeae*, which harbors chloroplasts that are most closely related to the chloroplasts in photoautotrophic euglenids, both possessed only the MAT paralogue. We found both the MAT and MATX paralogues in two photoautotrophic species (*Phacus orbicularis* and *Monomorphina pyrum*). The significant conflict between eukaryotic phylogenies inferred from MATX and SSU rDNA data represents strong evidence that MATX paralogues have undergone horizontal gene transfer across the tree of eukaryotes.

**Conclusions:**

Our results suggest that MATX entered the euglenid lineage in a single horizontal gene transfer event that took place after the secondary endosymbiotic origin of the euglenid chloroplast. The origin of the MATX paralogue is unclear, and it cannot be excluded that it arose by a gene duplication event before the most recent common ancestor of eukaryotes.

## Background

Methionine adenosyltransferase (MAT) is a cytosolic ubiquitous enzyme that synthesizes S-adenosyl-L-methionine (SAM), a molecule that is one of the most important metabolites in living cells. SAM serves as the major methyl donor to phospholipids, DNA, RNA and other small molecules and is the second most widely used enzyme substrate after ATP [1,2]. MAT is a well-conserved enzyme that is encoded in the genomes of most eukaryotes, eubacteria, and archaebacteria (which have a highly divergent version of the gene) and has been well studied at the primary, secondary, and tertiary structural levels [3-5]. Except for the mammalian MAT II, which is a hetero-oligomer [6], members of the MAT family are homo-oligomers that usually form tetramers consisting of four identical subunits; the two active sites are located between the subunits in each dimer [3]. Mammalian MAT III and archaeal MATs form dimers [7].

Multiple sequence alignments of MAT genes from a wide diversity of eukaryotes demonstrated a paralogue of MAT, named MATX, with distinctive features that are absent in all other eukaryotic MATs. These features include four specific insertions and a large number of unique substitutions [8]. The recombinant MATX from *Euglena gracilis* has been found to function as a homo-dimer with activities comparable to MATs from other eukaryotes [9]. Molecular phylogenetic analyses clearly showed that MATX is related to other eukaryotic MATs, but it forms a long branch in the eukaryotic subtree [8]. The majority of MATX paralogues occur in four distantly related groups of photosynthetic eukaryotes: haptophytes, photosynthetic euglenids, diatoms, and dinoflagellates. MATX was also detected in a pelagophyte alga *Aureococcus anophagefferens*[10]. All organisms possess either the MAT or the MATX form of the gene, with the exception of five diatom species that have both paralogues and *A. anophagefferens* that harbors two different homologues of MAT in addition to MATX [8,10].

A similar punctate distribution of two paralogues with the same function was reported for “elongation factor 1-alpha” (EF-1α) and its paralogue “elongation factor like” (EFL), which are highly conserved members of a GTPase superfamily involved in translation. Like MAT/MATX, the EF-1α/EFL paralogues have a patchy distribution across the tree of eukaryotes and rarely occur together in the same organism. EFL has been localized so far in eight groups of unrelated organisms: dinoflagellates, haptophytes, cercozoans, green algae, choanoflagellates, fungi, diatoms, and radiolarians [11-17].

The punctate distributions of MAT/MATX and EF-1α/EFL across the tree of eukaryotes can be explained by two scenarios: (1) a deep paralogy, whereby both paralogues were present in an ancient common ancestor followed by differential loss of one or the other paralogue in descendant lineages; and (2) a horizontal (syn., lateral) gene transfer (HGT), whereby a more recent origin of one paralogue (most likely the less frequent one, such as MATX) in one lineage of eukaryotes is followed by the spread of this paralogue to other distantly related lineages via horizontal transfer.

These scenarios differ in their assumptions. The first scenario hypothesizes coexistence and probably co-expression of both paralogues in one cell for a long time without negative effects on the organism. This scenario explains the distribution purely by vertical transmission. In this case, MATX must have originated by gene duplication from the MAT already present in the common ancestor of all MATX containing taxa. This organism was very ancient and not very distantly related, maybe identical, to the most recent common ancestor of eukaryotes. Since that time, MAT and MATX must have been propagated side by side in the genomes of the descendants to much more recent nodes of eukaryotic evolution and in some cases (diatoms) even to extant organisms.

The second scenario assumes that one (MATX) can be horizontally transferred and is capable of functional replacement of the MAT form soon after the transfer. Our previous work on the model systems of *Euglena gracilis* and *Trypanosoma brucei* indicates that MATX fulfills the assumptions for both of these scenarios, because this paralogue can be co-expressed with MAT and can immediately take over its function [18]. By contrast, EFL was capable of long-term co-expression, but was not able to functionally replace EF1-α. Based on these results, neither of the two evolutionary scenarios can be refuted for MAT/MATX. However, in the case of EF1-α/EFL, HGT is apparently more difficult and likely played a less important role in the evolutionary history of this paralogue couple [18].

There are several questions associated with the putative HGT explanation for the origin and distribution of the MATX paralogue that remain unanswered. For instance, under what circumstances would the highly divergent MATX evolve within one recent group of eukaryotes and in which lineage could it happen? One hypothesis posits that MATX evolved during a secondary endosymbiotic origin of plastids from the endosymbiont copy of the MAT gene, which was released from purifying selection and underwent accelerated sequence evolution [8]. Therefore, an analysis of the distribution of MAT/MATX in euglenids provides an opportunity to evaluate this possibility.

The Euglenida is a large group of marine and freshwater eukaryotic flagellates with diverse modes of nutrition, including phagotrophy, osmotrophy, photoautotrophy, and a recently discovered example of mixotrophy (a euglenid capable of both phagotrophy and photosynthesis) [19,20]. Photosynthetic and secondarily osmotrophic euglenids (i.e., colorless euglenids that have lost photosynthesis) form a monophyletic group that is the sister lineage to the mixotrophic *Rapaza viridis* and is nested within a paraphyletic assemblage of phagotrophic euglenids. It is inferred that the secondary chloroplast was gained through secondary endosymbiosis in the most recent common ancestor of all photosynthetic euglenids, including *R. viridis*[19-22]. The marine flagellate *Pyramimonas* (Pyramimonadales*,* Prasinophyta) is inferred to be the closest known relative of the euglenid chloroplasts (Turmel et al. 2009). In this study, we investigated the distribution of MAT and MATX in euglenids and *Pyramimonas* in order to evaluate whether the origin of MATX occurred simultaneously with the secondary endosymbiotic origin of the euglenid chloroplast. These data were also expected to provide insights into whether euglenids were the first group of eukaryotes to evolve the MATX paralogue.

## Results

### MAT and MATX phylogeny and distribution of MATX in euglenids

**Figure 1:**
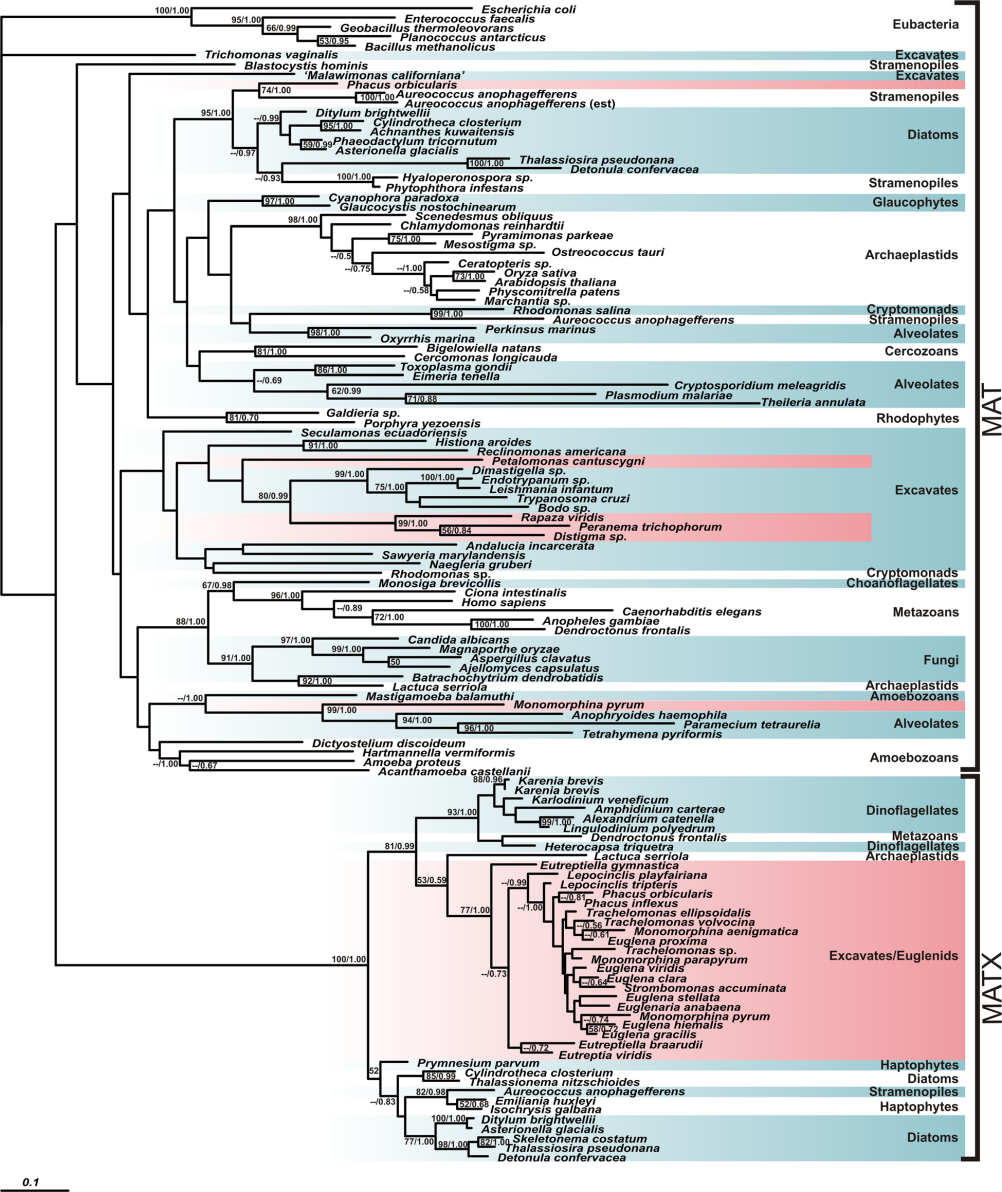
**Maximum likelihood phylogeny of MAT and MATX.** The tree was constructed by maximum likelihood method in RAxML from the 347 amino acid positions. The values at nodes represent maximum likelihood bootstraps/Bayesian posterior probabilities; only values above 50% and 0.5, respectively, are shown. Euglenid taxa are marked in red.

We generated six new sequences of MAT and 20 new sequences of MATX. The MAT sequences were obtained from heterotrophic euglenids (*Petalomonas cantuscygni* and *Distigma* sp.), the mixotroph *Rapaza viridis*, two photoautotrophic euglenids (*Phacus orbicularis* and *Monomorphina pyrum*) and the prasinophyte alga *Pyramimonas parkeae*. The MATX sequences were obtained from all investigated photoautotrophic euglenids, except *Rapaza viridis* (Table 1). The sequences retrieved from transcriptome projects were complete; sequences amplified from cDNA (*Pyramimonas parkeae, Trachelomonas* sp., *Distigma* sp*., Monomorphina aenigmatica* and *Monomorphina pyrum*) were partial (approximately 430 amino acids). We found additional so far unnoticed partial MATX homologues in GenBank from the haptophyte *Prymnesium*, the plant *Lactuca serriola* and the beetle *Dendroctonus frontalis*. Further database searches revealed that *Lactuca* and *Dendroctonus* also contain the MAT paralogue. The presence of the MATX paralogue in the single species of plant and metazoa is highly suspicious, and we treat this data with caution because we cannot exclude the possibility of contamination by foreign RNA in the *Lactuca* and *Dendroctonus* transcriptome data sets. The MAT sequences of *Rhodomonas* sp*., Rhodomonas salina, Thalassionema sp*. and *Peranema trichophorum* and the MATX sequence of *Karenia brevis* retrieved from GenBank were also incomplete. Despite their incompleteness, all MAT and MATX sequences were suitable for determining the paralogue type and for phylogenetic analyses; therefore, all sequences were added to the alignment with published MAT/MATX sequences for phylogenetic analysis (Figure 1).

**Table 1:** Sources of sequences applied in this study

**Taxon**	**Protein MAT/MATX**	**SSU**
*Euglena clara*	† supplement	AJ532423.1*
*Euglena stellata*	† supplement	AF150936.1*
*Euglena gracilis*	† supplement	AY029409.1*
*Euglena hiemalis*	† supplement	DQ140157.1*
*Euglena proxima*	† supplement	EU624027.1*
*Euglena viridis*	† supplement	AJ532415.1*
*Euglenaria anabaena*	† supplement	AF242548.1*
*Eutreptiella braarudii*	† supplement	AJ532397.1*
*Eutreptiella gymnastica*	▲ KF383289	▲ KF559331
*Distigma* sp.	▲ KF383287	
*Eutreptia viridis*	† supplement	AF157312.1*
*Lepocinclis tripteris*	† supplement	AF286210.1*
*Lepocinclis playfairiana*	† supplement	KF267871*
*Monomorphina aenigmatica*	▲ KF383291	AF283313.1*
*Monomorphina parapyrum*	† supplement	AF112874
*Monomorphina pyrum*	▲ KF383286 MAT ▲ KF383290 MATX	▲ KF559330
*Phacus inflexus*	† supplement	FJ719629.1*
*Phacus orbicularis*	† supplement	AF283315.1*
*Pyramimonas parkeae*	▲ KF383285	
*Rapaza viridis*	▲ KF383288	AB679269.1*
*Trachelomonas ellipsoidalis*	† supplement	DQ140135.1*
*Trachelomonas* sp.	▲ KF383292	AJ532447.1*
*Trachelomonas volvocina*	† supplement	AF096995.1*
*Strombomonas accuminata*	† supplement	EU624029.1*

The sequences downloaded from GenBank are marked by *; sequences obtained by Sanger sequencing method in this study are marked by ▲, sequences obtained from transcriptome projects sequenced by Roche 454 sequencing were marked by † and are available in supplement.

In the phylogenetic tree (Figure 1), MATX paralogues formed a well-supported clade that was separated from the MAT paralogues by a long stem. The tree was rooted by five bacterial outgroups within the MAT paralogues, with *Trichomonas vaginalis* MAT being the most basal branch. However, the backbone topology of the MAT tree was weakly supported, and the MATX branch was situated only one node apart from prokaryotes. We used Kishino Hasegawa (KH), weighted KH (WKH), Shimodaria Hasegawa (SH) and weighted SH (WSH) tests to evaluate whether the root position between MAT and MATX paralogues is significantly worse than the suggested root on the *T. vaginalis* branch. The tests showed that this root position cannot be excluded (p = 0.076 for KH and WKH, p = 1.00 for SH and p = 0.945 for WSH).

The MATX sequences from photoautotrophic euglenids formed a well-supported subclade (bootstrap 77%) within the more inclusive MATX clade and branched as the sister group to a clade consisting of *Lactuca*, dinoflagellates and *Dendroctonus*. The MAT sequences from the heterotrophic euglenids clustered together with kinetoplastids; the MAT sequence from *P. parkeae* branched together with other green algae; and the MAT sequences from *M. pyrum* and *P. orbicularis* clustered with ciliates and *Aureococcus*, respectively.

**Figure 2:**
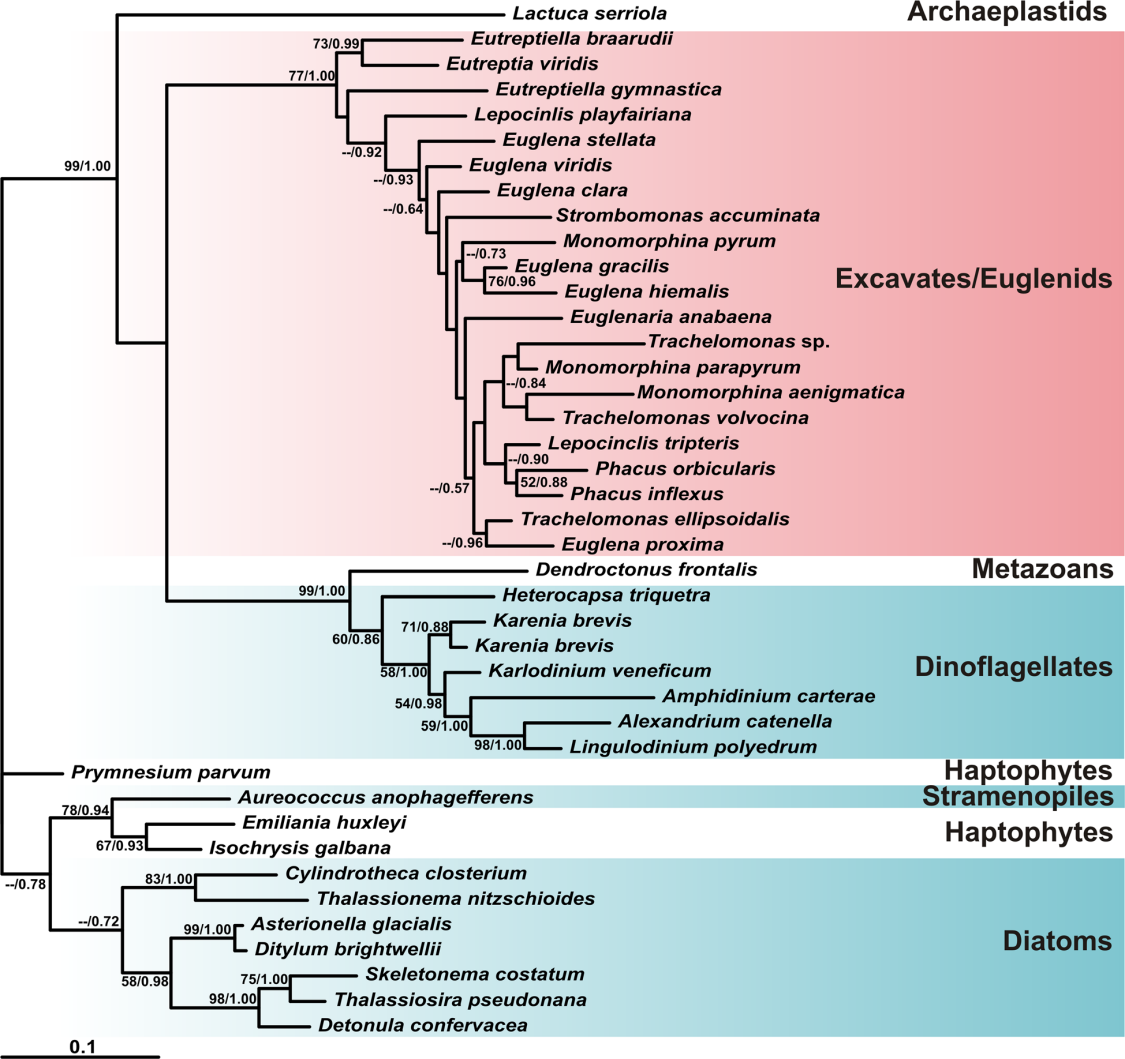
**Maximum likelihood phylogeny of MATX clade.** The tree was constructed by maximum likelihood method in RAxML from the 392 amino acid positions. The values at nodes represent maximum likelihood bootstraps/Bayesian posterior probabilities; only values above 50% and 0.5, respectively, are shown.

We also performed an independent analysis of MATX sequences that enabled us to use more alignment positions to reconstruct the phylogenetic relationships within the MATX clade (Figure 2). The tree was rooted with the branch of diatoms, haptophytes and *Aureococcus* according to Figure 1.

### Comparison of MATX and SSU rRNA gene phylogeny

We investigated whether or not the phylogeny of the MATX paralogues differs significantly from the species phylogeny. Significant differences would indicate that MATX has not evolved vertically but instead experienced HGTs between the MATX containing taxa. As “species trees”, we have used topologies inferred from small subunit (SSU) rRNA gene sequences and also manually constructed topologies reflecting current view of species relationships. The SSU rRNA gene tree and manual species topologies differed in minor details and they are reported in Additional file 1 and in Additional file 2: Figure S1 and Additional file 3: Figure S3. We used the KH and SH tests to compare the species topologies with the best MATX topology and the set of 500 bootstrap topologies calculated from MATX alignment (Table 2). The tests showed that the “species topologies” are strongly rejected (p value = < 7*10^-6^). To be sure that the conflict with the SSU rRNA gene tree topology is not caused only by the *Lactuca*, *Dendroctonus* and *Aureococcus* MATX sequences, whose origin is dubious, and *Prymnesium*, the sequence of which is very incomplete, we repeated the tests after exclusion of these four taxa. The “species topologies” were again rejected (p = < 2*10^-4^). The “species topologies” were significantly excluded also if we compared topologies rooted by *Trichomonas* and *Escherichia*, although the significance was lower (p = < 0.001).

**Table 2:** Results of topology tests

	**KH**	**WKH**	**SH**	**WSH**
**MATX (1)**	**0/0**	**0/0**	7*10^**-6**^**/0**	**0/0**
**MATX excl. APLD (2)**	**0/0**	**0/0**	1*10^**-4**^/2*10^**-4**^	5*10^**-5**^/4*10^**-6**^
**MATX rooted (3)**	8*10^**-6**^**/0**	1*10^**-5**^**/0**	0.001/2*10^**-5**^	1*10^**-4**^**/0**
**MATX rooted exl. APDL (4)**	**0/0**	**0/0**	**0.001/0.001**	2*10^**-4**^/2*10^**-4**^
**MATX euglenids (5)**	0.004/0.004	0.003/0.003	0.25/0.246	0.209/0.172

The p-values of significance for differences between likelihoods of MATX gene tree vs. likelihoods of species trees. In each cell are given p-values using species tree inferred from phylogeny of SSU rRNA/species tree based consensus from a literature. The tests were performed for five sets of taxa: (1) full MATX data set, (2) MATX excluding *Aureococcus*, *Prymnesium*, *Lactuca* and *Dendroctonus* (excl. APLD), (3) rooted full MATX data set, (4) rooted MATX excl. APLD and (5) MATX of euglenids. Four tests were used: Kishino Hasegawa (KH), weighted Kishino Hasegawa (WKH), Shimodaria Hasegawa (SH), weighted Shimodaria Hasegawa (WSH). P-values = < 0.001 are given in bold.

Similarly we compared the MATX topology (Additional file 4: Figure S2) with the SSU rRNA gene tree (Additional file 3: Figure S3) and manual species topologies (Additional file 1) of the subclade of photosynthetic euglenids. In this case, the tests showed that the euglenid “species topologies” cannot be rejected (p > = 0.003).

## Discussion

### Distribution of MAT and MATX paralogues in euglenids

**Figure 3:**
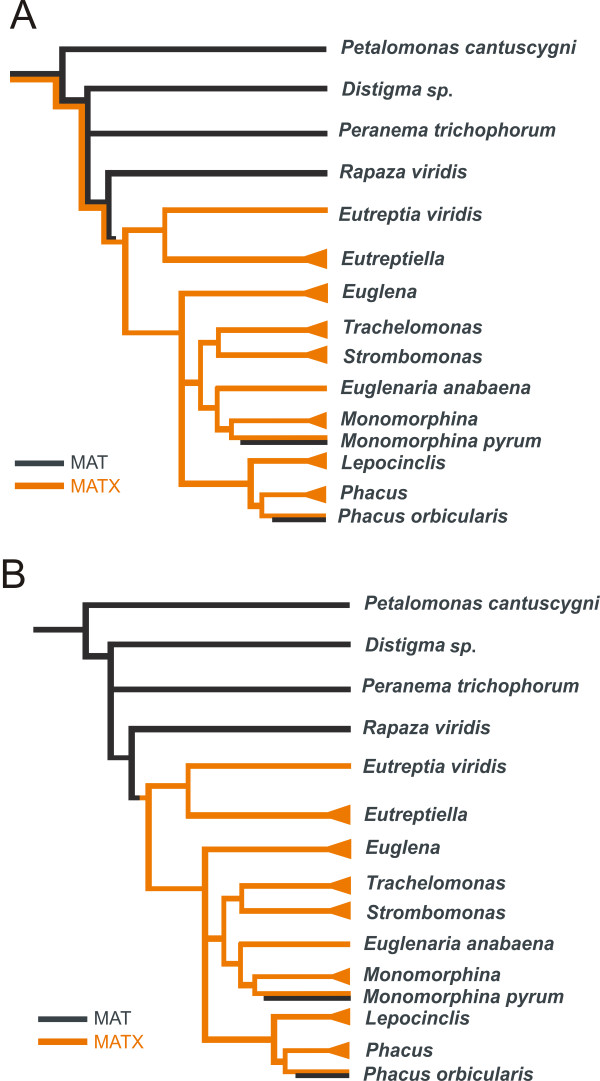
**Schematic trees illustrating two possible scenarios of MAT/MATX evolution mapped on the currently accepted phylogenetic relationships of euglenids.** The presence of MATX is marked with orange color and MAT is colored with black. **(A)** Scenario involving deep paralogy followed by differential losses. **(B)** Scenario involving horizontal gene transfer.

Some genes are dispersed across the tree of eukaryotes in a punctate pattern, which means that they are present in unrelated taxa and absent in interspersed lineages. This observation suggests that the evolution of these genes was complicated and may involve events like gene duplications (the origin of paralogues), horizontal gene transfers, and gene losses. Deciphering the history of such a gene is often difficult. Two of the most enigmatic examples are (1) elongation factor 1-alpha (EF-1α) and its paralogue elongation factor-like (EFL) and (2) methionine adenosyl transferase (MAT) and its paralogue MATX [8,11]. In both cases, these essential genes come in two paralogues that exhibit a patchy distribution among eukaryotes and are mutually, almost strictly, exclusive in their occurrence. We considered two scenarios to explain the possible evolution of the distribution of MAT and MATX: (A) a deep paralogy scenario and (B) a horizontal gene transfer scenario. MAT and MATX gene histories in euglenids according to these two scenarios are shown in Figure 3.

We detected MATX only in photoautotrophic euglenids. *Rapaza viridis*, which contains secondary chloroplasts and represents the earliest diverging lineage within the photoautotrophic clade, apparently possesses only the MAT form of the gene; the same holds for the heterotrophic euglenids (*Petalomonas*, *Distigma* and *Peranema*) and *Pyramimonas parkeae*, which contains the closest known relative of the euglenid chloroplast. Therefore, our results suggest that MATX is specific for the clade of photoautotrophic euglenids after the split of *Rapaza*. We also found two exceptions within the clade of photoautotrophic euglenids; *P. orbicularis* and *M. pyrum* both possess the MAT and MATX paralogues in their cDNAs, so both genes are transcribed in these species. The MATX form in these two species is located within the MATX clade with other photoautotrophic euglenids, while the MAT form is unrelated to euglenid MATs; the MAT of *P. orbicularis* branches together with the MAT sequences from *Aureococcus*, and the MAT in *M. pyrum* branches together with the MAT sequence from ciliates. These facts are most likely explained by two independent horizontal gene transfers of MATs from two different sources into two different lineages of euglenids.

### Evolution of the MAT and MATX paralogues

We will focus on how well the observed data fit within the context of the two alternative hypotheses for the evolution of MAT and MATX in euglenids in particular and eukaryotes in general: (A) the deep paralogy scenario and (B) the horizontal gene transfer scenario (Figure 3). Let us first suppose that the deep paralogy scenario (Figure 3A) is correct. This scenario requires at least four independent losses of the MATX gene to explain its distribution in euglenids and many more losses of MATX to explain its distribution within the tree of eukaryotes. Gene losses are frequent events and many losses are not in themselves unlikely. Slightly suspicious, however, is the discrepancy in the number of MAT losses versus the number of MATX losses in this scenario. MAT was lost in euglenids (and within the Euglenozoa) only once, while MATX was lost at least four times only within euglenids. A similar disproportion of losses is present in the tree of eukaryotes. If we compare the MAT/MATX history to the case of EF-1α/EFL, the discrepancy is not as significant in the EF-1α/EFL case; the occurrence of EFL is more fragmented not only in euglenids but also in other eukaryotic groups [15-17,23]. To our knowledge, it is impossible to evaluate the significance of the observed disproportion between the number of losses of one paralogue compared to the other, so we must conclude that in this respect our observations do not contradict the deep paralogy scenario.

Moreover, if the deep paralogy scenario is correct (Figure 3A), then we would expect both paralogues MAT and MATX to be present in the most recent common ancestor of all MATX-containing taxa, which is likely identical to the most recent common ancestor of eukaryotes. If so, then we would expect that the root of the tree in Figure 1 will be positioned between the MAT and MATX lineages. This is true for EF-1α/EFL tree [11]. In the case of MAT/MATX, the bacterial outgroups form the sister branch to MAT of *Trichomonas vaginalis*, and the MATX clade is positioned within the MAT lineages. However, the bootstrap values supporting the backbone of the MAT/MATX tree are very low (Figure 1), and the root position on the MATX branch was not rejected by the statistical tests. In this respect our data do not contradict the deep paralogy scenario.

The deep paralogy scenario also assumes that the two paralogues can be co-expressed together in one organism. The observation that the two paralogues are simultaneously present in the transcriptomes of two different euglenids (*P. orbicularis* and *M. pyrum*), five diatoms, and *Aureococcus*[10] demonstrates that this is indeed possible. Moreover, we have confirmed this fact experimentally on the model system of *Euglena gracilis* and *Trypanosoma brucei*[18]. In this respect the data do not contradict the deep paralogy scenario.

Finally, the deep paralogy scenario expects that the relationships between the eukaryotic groups in the MATX part of the tree will correspond to the accepted eukaryotic phylogeny, because the gene, despite being lost in many lineages, has evolved vertically. This is apparently not true, because MATX sequences in dinoflagellates form a relatively robust sister branch to MATX sequences in euglenids (bootstrap = 81%), even though dinoflagellates are in fact more closely related to apicomplexans, ciliates, stramenopiles (including diatoms) and haptophytes. More importantly, the conflict between the global MATX phylogeny and the species phylogeny of the MATX containing taxa was significant in statistical tests. Within the clade of photoautotrophic euglenids, the MATX phylogeny also differed from species tree, but this difference was not significant. In this last respect, therefore, our data do contradict the scenario of deep paralogy followed by differential losses in its purest form. In order to explain this observation, we must invoke either horizontal gene transfers within the MATX clade or at least two more gene duplications and subsequent differential losses of putative paralogues within the MATX clade. The latter case would assume that some ancestral organisms would harbor at least four paralogues of this enzyme, which is inconsistent with the observation that most extant species contain only one paralogue (see Additional file 1); therefore, we conclude that MATX has not evolved vertically.

Let us now suppose that the horizontal gene transfer scenario is correct. The first assumption of this scenario is that the MATX paralogue is capable of horizontal transfer. The ability of the MATX paralogue to substitute the function of MAT has been proven experimentally in *E. gracilis* and *T. brucei*[18]. In this study, we have also revealed two relatively clear cases of MAT horizontal transfers from different sources into *P. orbicularis* and *M. pyrum*. In order to explain the distribution of MATX in euglenids through HGT, we only require a single horizontal gene transfer shortly after *Rapaza viridis* split from the other photoautotrophic euglenids (Figure 3B); only a few more horizontal gene transfers would be necessary to explain the distribution of MATX in all eukaryotes. Taken together, the data suggests that MATX is capable of HGT and the number of required events is low. In this respect, the data do not contradict the horizontal gene transfer scenario.

The second assumption of the HGT scenario is that there was a eukaryotic group in which the MATX first evolved and then subsequently spread into other lineages of eukaryotes. Such a group would ideally appear as a paraphyletic assemblage near the very base of MATX clade. At the same time, the root of the MAT/MATX tree would be situated inside the MAT paralogues. The data collected so far do not suggest any source group, because the taxa with MATX either form monophyletic groups (e.g., euglenids and dinoflagellates) or have unclear phylogenetic positions (e.g., diatoms, haptophytes and *Aureococcus*). Our working hypothesis that the MATX originated during the secondary endosymbiotic origin of the euglenid chloroplast ([8]) is not supported by the fact that the MATX paralogue is absent in both *Rapaza viridis* and the closest relative of the euglenid chloroplast, *Pyramimonas*. Moreover, the MATX paralogues in euglenids do not form a paraphyletic group, but instead form a robust clade within the more inclusive MATX clade. The position of the root between MAT and MATX lineages cannot be rejected, and both paralogues might have been present in the common ancestor of all eukaryotes. The current data are in this respect not in direct conflict but, at the same time, they are also not supportive of the horizontal gene transfer scenario.

## Conclusions

Our data are not entirely consistent with either of the two scenarios for MAT/MATX evolution in their purest forms. The hypothesis of deep paralogy followed by differential losses is rejected by the fact that MATX did not evolve purely by vertical transmission. The hypothesis of a more recent origin of MATX followed by spread via horizontal gene transfers is complicated by the absence of a source of the first MATX paralogue and the fact that both paralogues could be present in the most recent common ancestor of all eukaryotes. Therefore, we infer that the MATX paralogue spread among eukaryotes via HGT; however, the original source of MATX is not yet known and it could originate by gene duplication from MAT in the last eukaryotic common ancestor.

We also infer that euglenids were not the group in which the MATX paralogue evolved. Instead, a foreign MATX paralogue substituted the ancestral euglenid MAT paralogue in a single horizontal gene transfer event that occurred after the secondary endosymbiotic origin of the euglenid chloroplast (Figure 3B). Although the donor of the euglenid MATX paralogue is not known, the MATX paralogue, once established, may have evolved vertically within the clade of photoautotrophic euglenids. Two photoautotrophic euglenids (*P. orbicularis* and *M. pyrum*) regained a new version of the MAT paralogue by recent horizontal gene transfers from two different eukaryotic lineages and now contain both paralogues. Overall, the case study of MAT/MATX illustrates the complex evolutionary histories of some eukaryotic genes and highlights the prevalence of gene duplications, differential losses of paralogues, and horizontal gene transfer events during the course of eukaryotic evolution.

## Methods

### Euglenid strains and culture conditions

All cultures used in this study are listed in Table 1. Strains of *Eutreptiella gymnastica* (SCCAP K-0333), *Trachelomonas* sp. (SCCAP K-1380) and *Pyramimonas parkeae* (SCCAP K-0007) were obtained from the Scandinavian Culture Collection of Algae and Protozoa (SCCAP). Strains of *Monomorphina pyrum* (CCAP 1261/4B) and *Monomorphina aenigmatica* (CCAP 1261/9) were obtained from the Culture Collection of Algae and Protozoa (CCAP). *Distigma* sp. was isolated from samples collected from freshwater sediment from Czech Republic (50°27’N, 13°20’E). This culture was not monoeukaryotic and contained various other protists, therefore, we used a method of single cell cloning by serial dilution to obtain a monoclonal *Distigma* sp. culture. *Rapaza viridis* was isolated and cultured from marine sediment samples from Canada (48° 47.551’ N, 125° 06.974’ W) [20]. *Euglena clara* (SAG 25.98), *Euglena gracilis* (SAG 1224-5/25), *Euglena proxima* (SAG 1224-11a), *Eutreptia viridis* (SAG 1226-1c), were obtained from the Culture Collection of Algae at Goettingen, Germany. *Euglena stellata* (UTEX 372), *Trachelomonas volvocina* (UTEX 1327), *Monomorphina parapyrum* (UTEX 2354) and *Euglenaria anabaena* (UTEX 373) were obtained from the Culture Collection of Algae at the University of Texas, Austin Texas, USA. *Euglena viridis* (ATCC PRA110) was from the American Type Culture Collection, Manassas, Virginia, USA and *Eutreptiella braarudii* (CCMP 1594) was obtained from the National Center for Marine Algae and Protozoa, East Boothbay, Maine, USA. *Phacus inflexus* (ACOI 1336) and *Phacus orbicularis* (ACOI 996) were obtained from the Coimbra Collection of Algae, Coimbra, Portugal. Culture of *Petalomonas cantuscygni* (CCAP 1259/1) was provided by Dr. Mark Farmer at the University of Georgia, Athens, Georgia, USA and it was originally obtained from the Culture Collection of Algae and Protozoa. *Strombomonas accuminata* NJ, S 716 and *Trachelomonas ellipsoidalis* NJ, ST1 are cultures maintained in the Triemer lab which were originally isolated from pond samples from New Jersey, USA; *Lepocinclis tripteris* MI 101 and *Lepocinclis playfairiana* MI 102 are cultures isolated from ponds near Michigan State University, East Lansing, MI, USA.

### DNA, RNA isolation and preparation of cDNA

Genomic DNA from *Eutreptiella gymnastica*, *Trachelomonas* sp., *Pyramimonas parkeae*, *Monomorphina pyrum, Monomorphina aenigmatica,* and *Distigma* sp. was extracted from strains using the Qiagen Blood and Tissue kit and total RNA was isolated from 150 ml of well-grown cultures (approx. 25*10^6^ cells) using TRIzol Reagent (Invitrogen). Total RNA from *Rapaza viridis* was isolated using Ambion® RNAqueous-Micro Kit (Life technologies). mRNA was purified from total RNA with the use of Dynabeads mRNA Purification Kit (Invitrogen). cDNA was then prepared using Smarter PCR cDNA Synthesis Kit (Clontech) according to the manufacturer’s protocol with 15 to 27 cycles of cDNA amplification (depending on the amount of mRNA used in the first-strand synthesis).

In case of *E. gracilis, M. parapyrum, S. accuminata* and *L. playfairiana* the total RNA was extracted by grinding wet biomass in liquid nitrogen followed by purification using RNA/DNA Maxi Kit (Qiagen); mRNA, whenever used for cDNA synthesis, was purified from total RNA using Qiagen Oligotex mRNA Maxi Kit. cDNA was prepared using Smart (later Smarter) cDNA synthesis Kit (Clontech) or by similar technology provided by MINT cDNA synthesis Kit (Evrogen). cDNA libraries were normalized using Trimmer cDNA normalization Kit (Evrogen). The resulting normalized cDNA was adapted for Roche 454 sequencing by performing a multiple last amplification step, pooling the PCR products in order to achieve the overall amount of cDNA acceptable for sequencing.

For the remaining euglenid strains, total RNA was isolated using RNAzol RT RNA Isolation Reagent (Molecular Research Center, Inc.). High level purification of total RNA was achieved using MEGAclear Kit (Ambion). Next, mRNA was isolated using MIcroPoly(A)Purist Kit (Ambion). Preparation of cDNA suitable for the next generation sequencing was according to cDNA Rapid Library Preparation Manual (Roche, GS FLX Titanium Series, later GS FLX + Series - XL+).

### Amplification, sequencing and assembly

In case of *Pyramimonas parkeae, Eutreptiella gymnastica, Trachelomonas* sp., *Distigma *sp., *Monomorphina aenigmatica* and *Monomorphina pyrum* we have amplified the MAT or MATX genes from cDNA template using slightly modified primers of Kamikawa et al. [10]: Forward primer MATA3-F (5’-GAGYMMGTSAVYGARGGYCAYCCXGACAA-3‘) directed at the consensus amino acid (aa) sequence GHPDK and the reverse primer MATB3-R (5’-CCRTGNGCNCCCCADCCDCCRTAXGT-3’) directed at the eukaryotic consensus aa sequence TYGGWGAH inside a conserved block. Amplification was carried out in 25-μl reactions with 1.5 μl of the diluted cDNA as a template using EmeraldAmp MAX PCR Master Mix (TaKaRa Bio Inc.) and the following program: a hot start at 95°C for 4 min, followed by 35 cycles of denaturation at 95°C for 30 s, annealing at 55°C for 60 s and extension at 72°C for 90 s, finishing with an extension at 72°C for 15 min. The PCR products were excised from the gel, cloned into pGEM-T Easy Vector System (Promega) and sequenced. The new sequences were deposited in GenBank under the accession numbers listed in Table 1.

Small subunit (SSU) ribosomal RNA gene from *E. gymnastica* was amplified from genomic DNA with “universal” eukaryote SSU primer pairs Medlin A (5’-CTGGTTGATCCTGCCAG-3‘), Medlin B (5’-TGATCCTTCTGCAGGTTCACCTAC-3’) described by Medlin et al. [24]. Amplification was carried out using the following program: a hot start at 95°C for 4 min, followed by 35 cycles of denaturation at 95°C for 30 s, annealing at 55°C for 60 s and extension at 72°C for 90 s, finishing with an extension at 72°C for 15 min. Medlin A, Medlin B, EPA-23 (5’- GTCATATGCTTYKTTCAAGGRCTAAGCC -3’), EPA-2286 (5’- TCACCTACARCWACCTTGTTACGAC -3’) according to Müllner et al. [25] and our primers SSU 633-F (5’- GGCAGCAGGCRCGCAAATTGC -3’) and SSU 2031-R (5’- TCAACCAGACAAATCACTYCACCAA -3’) were used for sequencing of PCR products.

Small subunit (SSU) ribosomal RNA gene from *L. playfairiana* and *M. parapyrum* was amplified from genomic DNA with nuclear SSU primers 18S_1A (AAYCTGGTTGATCCTGCCAGT) and 18S_1520B (TGATCCTTCTGCAGGTTCACCTAC). Amplifications were carried out using 5 min of denaturation at 94°C and 30 cycles of the following: 94°C for 30 s, 45°C – 50°C for 1 min, 72°C for 2 min, a final extension at 72°C for 11 min. For sequencing of PCR products were used primers 18S_1A, 18S_1520B, 18S_300F (WGGGTTYGATTCCGGAG), 18S_528F (CGGTAATTCCAGCTCC), 18S_516R (ACCAGACTTGCYCTCC), 18S_960F (TTTGACTCAACRCGGG) and 18S_1055R (CGGCCATGCACCACC).

For the 454 sequences obtained from cDNAs, the raw reads (SFF File format) from 454 were filtered to remove reads shorter than 50 bp and all reads which had more than 30% of the bases with a Phred quality score less than 30 using NGS QC TK [26] were excluded. The resulting high quality reads were assembled using Roche's proprietary "Newbler" software version 2.6 with "cDNA" option. Assembled contigs shorter than 200 bp were excluded.

The full length of euglenid MATX genes were 1290 bp. Some of the sequences were incomplete: *P. orbicularis* (length 1257 bp), *M. pyrum* (length 906 bp), *M. aenigmatica* (length 843 bp), *Trachelomonas* sp. (length 909 bp) and *E. anabaena* (length 1266 bp). The length of the MAT genes were 1167 bp for *P. cantuscygni*, 1137 for *P. orbicularis*, 795 bp for *R. viridis*, 774 bp for *M. pyrum*, 765 bp for *Distigma* sp. and 720 bp for *P. parkeae*.

### Phylogenetic analyses

The MAT and MATX protein sequences were aligned in ClustalX [27], the SSU rRNA gene sequences were aligned in MAFFT (http://www.genome.jp/tools/mafft/) using G-INS-I option [28]. The alignments were manually refined in BioEdit 7.0.5.3. [29]. The regions, which could not be unambiguously aligned, were excluded from the analyses.

A phylogeny of eukaryotic MAT and MATX was inferred from 123 sequences using 347 aligned amino acid positions; the phylogenetic relationships within the MATX clade were inferred from 41 sequences and 405 positions; the phylogenetic relationships within the euglenid subgroup of the MATX clade were inferred from 21 sequences and 399 alignment positions. Maximum likelihood trees were estimated by RAxML_HPC version 2.3.3 [30] using the best fitting models as determined by Prottest (http://darwin.uvigo.es/software/prottest2_server.html) [31] and 10 replicates of starting tree construction. The models were PROTGAMMALG for MAT + MATX and MATX of euglenids and PROTGAMMAWAG for analysis of eukaryotic MATX clade. Bootstrap supports (BS) were calculated from 500 replicates. Bayesian trees were estimated by MrBayes version 3.1.2 (Ronquist and Huelsenbeck 2003) using the WAG + GAMMA + Invariants + covarion model of substitution. In case of MAT + MATX analysis (Figure 1), two MCMC were run for 5 860 000 generations, trees from the first 1000 000 generations were discarded as burn-in. In case of MATX analysis (Figure 2), two MCMC were run for 17 775 000 generations, trees from the first 2 818 500 generations were discarded as burn-in.

For the purposes of topology testing, pruned and rooted data sets of MATX clade were analyzed – 40 sequences (only one *Karenia brevis* sequence was used), 36 sequences (without *Aureococcus*, *Prymnesium*, *Dendroctonus* and *Lactuca*) and both previous data sets rooted by *Trichomonas* and *Escherichia* (i.e. 42 and 38 sequences). All alignments contained 405 amino acid positions and were analysed as described above. Phylogenetic trees of SSU rDNA were inferred by maximum likelihood method from the corresponding set of taxa – 40 and 36 sequences in unrooted, 42 and 38 sequences in rooted analyses of MATX clade and 21 sequences of MATX containing euglenids. Unrooted and rooted SSU alignments contained 1525 and 1282 positions respectively. A maximum likelihood trees were estimated by RAxML_HPC version 2.3.3 [30] using the GTRGAMMA model of nucleotide substitution, 10 replicates of starting tree construction and BS were calculated from 500 replicates.

All data sets and trees generated in this study have been deposited in TreeBASE (study accession number is 15062).

### Topology testing

The Kishino Hasegawa (KH) [32] and Shimodaria Hasegawa tests [33] implemented in Consel 0.1j [34] were used for topology testing. We have decided not to report the results of approximately unbiased test [35] because we have realized that the test behaves very unstably for our data sets; re-testing of the same data sets produced very different p-values that sometimes differed in significance. Regarding the significance or non-significance at the p = 0.001 level, the results of the AU tests were in agreement with the results of KH and SH tests in most cases; however due to their instability, we have decided to report only the results of KH and SH tests.

A set of 503 topologies was created in order to test whether the relationships between MATX paralogues are in conflict with the relationship of MATX containing taxa as inferred from SSU rDNA sequences. This set of topologies contained the best topology inferred from an analysis of the MATX protein alignment by RAxML, 500 topologies from bootstrap permutations of the MATX alignment generated by RAxML, the best tree inferred by RAxML from the SSU rRNA alignment of the same set of taxa, and the manually constructed topology reflecting the current view of species relationships. The latter two topologies representing species trees are given in Additional file 1 and in Additional file 2: Figure S1 and Additional file 3: Figure S3. Site likelihoods for topologies 1–501 were inferred by Treepuzzle 5.2. [36] using MATX gene alignment, WAG + I + Γ model of amino acid substitution and parameter values inferred from the topology nr. 1. Site likelihoods for topologies 502 and 503 were inferred by Treepuzzle using MATX gene alignment, WAG + I + Γ model of amino acid substitution and parameter values inferred from these topologies. The sets of site likelihoods were then compared by the KH, weighted KH (WKH), SH and SH (WSH) test in Consel 0.1j [34]. The tests were performed for (1) the full set of MATX paralogues from 40 taxa, (2) a set of MATX paralogues, excluding MATX from *Aureococcus*, *Prymnesium*, *Dendroctonus* and *Lactuca*, (3) data set 1 rooted by *Trichomonas* and *Escherichia*, (4) data set 2 rooted by *Trichomonas* and *Escherichia*, and (5) a set of MATX paralogues from euglenids.

The same tests were used to evaluate whether or not the root position between MAT and MATX paralogues can be rejected. For these tests, we used topology shown in Figure 1, 500 bootstrap topologies calculated from the same alignment, and a topology that differed from Figure 1 only in the position of prokaryotic outgroups that were moved on the branch separating MAT and MATX paralogues. The tests were performed as described above.

### Availability of supporting data

All the supporting data are included as additional files.

## Competing interests

The authors declare that they have no competing interests.

## Authors’ contributions

JS participated on cDNA preparation (for *Distigma* sp., *P. parkeae*, *E. gymnastica*, *Trachelomonas* sp., *M. pyrum*, *M. aenigmatica* and *R. viridis*), data analysis, in the sequence alignments and drafted the manuscript. NY provided *Rapaza viridis* RNA and revised the manuscript. BSL revised the manuscript. RET provided the transcriptome data for the rest of euglenid species and revised the manuscript. VH supervised the study, performed the phylogenetic analyses and helped to draft the manuscript. All authors read and approved the final manuscript.

## Supplementary Material

Additional file 1 **Reconciliation of MATX gene tree with species tree.** We have used the software Jane (http://www.cs.hmc.edu/~hadas/jane/) to reconcile the MATX gene tree with the species tree. For this analysis we have excluded taxa with very incomplete sequence (*Prymnesium*) or taxa, whose MATX sequences could be result of contamination (*Lactuca* and *Dencroctonus*). If we set the cost of gene loss to 0, which could be a realistic value in case of loss of one of two paralogues, then the discrepancy between MATX gene tree and species tree can be explained by the same number of events if we consider duplications and differential losses (A) or horizontal gene transfers (B). Click here for file

Additional file 2: Figure S1 Maximum likelihood phylogeny of MATX containing taxa based on SSU rRNA gene. The tree was constructed by maximum likelihood method in RAxML from the 1525 nucleotide positions. The values at nodes represent maximum likelihood bootstraps, only values above 50% are shown. Click here for file

Additional file 3: Figure S3 Maximum likelihood phylogeny of MATX containing euglenid taxa based on SSU rRNA gene. The tree was constructed by maximum likelihood method in RAxML from the 1525 nucleotide positions. The values at nodes represent maximum likelihood bootstraps, only values above 50% are shown. Click here for file

Additional file 4: Figure S2 Maximum likelihood phylogeny of euglenid MATX. The tree was constructed by maximum likelihood method in RAxML from the 399 amino acid positions. The values at nodes represent maximum likelihood bootstraps, only values above 50% are shown. Click here for file
