# Supplementary material for: The evolution of paralogous enzymes MAT and MATX within the Euglenida and beyond
Source: BMC Evol Biol. 2014 Feb 11;14:25. doi: 10.1186/1471-2148-14-25 (PMC3923989; doi:10.1186/1471-2148-14-25)
Supplement: Additional file 1 — Reconciliation of MATX gene tree with species tree. We have used the software Jane (http://www.cs.hmc.edu/~hadas/jane/) to reconcile the MATX gene tree with the species tree. For this analysis we have excluded taxa with very incomplete sequence (Prymnesium) or taxa, whose MATX sequences could be result of contamination (Lactuca and Dencroctonus). If we set the cost of gene loss to 0, which could be a realistic value in case of loss of one of two paralogues, then the discrepancy between MATX gene tree and species tree can be explained by the same number of events if we consider duplications and differential losses (A) or horizontal gene transfers (B). [file 1471-2148-14-25-S1.docx]

**SUPPLEMENTARY DATA**

**Reconciliation of MATX gene tree with species tree**

We have used the software Jane (http://www.cs.hmc.edu/~hadas/jane/) to reconcile the MATX gene tree with the species tree. For this analysis we have excluded taxa with very incomplete sequence (Prymnesium) or taxa, whose MATX sequences could be result of contamination (Lactuca and Dencroctonus). If we set the cost of gene loss to 0, which could be a realistic value in case of loss of one of two paralogues, then the discrepancy between MATX gene tree and species tree can be explained by the same number of events if we consider duplications and differential losses (A) or horizontal gene transfers (B).

A


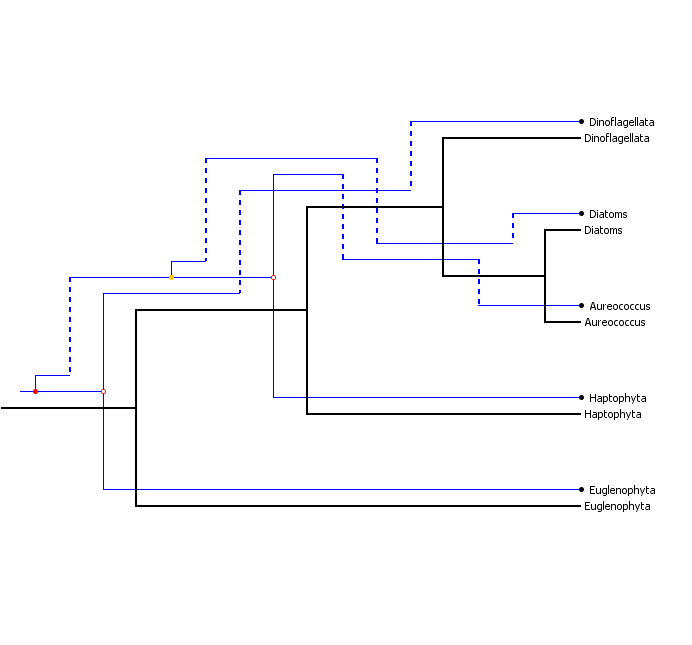


B


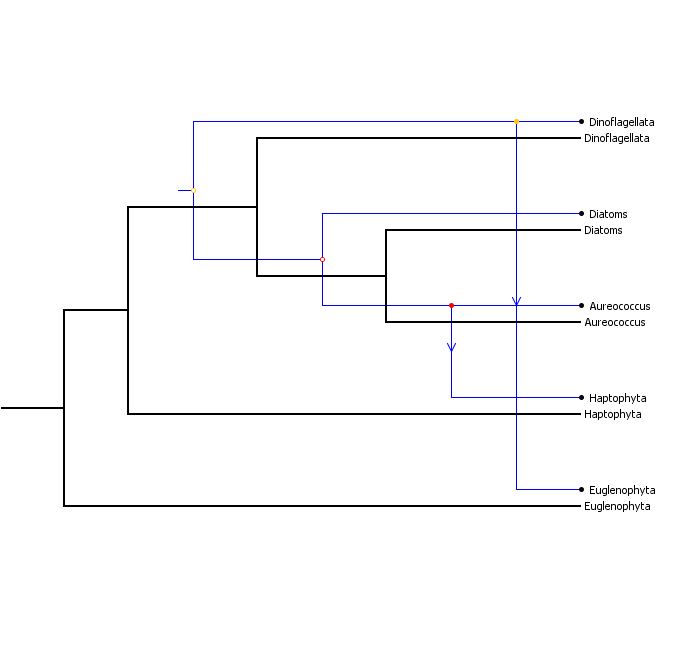


Although we cannot objectively reject alternative (A), we consider it less likely. Note that this alternative expects that the ancestors of dinoflagellates and stramenopiles would harbour four paralogues of the enzyme (three paralogues of MATX and one paralogue of MAT) over a long period of evolutionary time (marked by curly brace). This is unrealistic in our opinion because most extant species contain only one paralogue.

**MAT and MATX genes (translated proteins) of euglenids assembled from transcriptome projects.**

>Euglena clara methionine adenosyltransferase (MATX) mRNA, partial CDS

GAGTTCCTTTTCTCCTCTGAGTCAGTCAACGAGGGTCATCCCGACAAGCTCTGTGATCAGGTCTCTGATTCTGTGCTGGACGCATGTTTGACTGAGGACCCCAAGAGCAAGGTGGCCTGTGAGACAGCTGTCAAGGACAACATGGTTATGGTTCTGGGCGAGATCACCACCCAAGCTAAGCTGGATTATGACGCCATTGTGCGCAAGGCCGTTCGAAACATTGGCTTTGACTCCTTCGTAGATGACTTGTCCAGCGTGGATTCCAAGGGCCTGAGCTGTGACGACTGTGAGGTGTTGGTGCGAATCAACAAGCAATCCCCAGACATTGCTGGCGGTGTGCACGTTGGGCGTGATGAGATGGATTTGGGAGCTGGTGATCAGGGCATTATGTTTGGATATGCCTCTGATGAGACCCAGAGCACCATGCCCCTTACCCATTACCTCGCCACTAGGCTTGGCAAGACCCTCACCGACGTCCGCAAAGATGGCTCCCTCTGGTGGCTCCGTCCCGACGGCAAGACCCAGGTGACCATCAAGTACAAGCAGTGCCCCGATGGCTCTGTGGAGCCCCTGCTGATTCACACAGTGTTGATCTCCACGCAGCACTGCGAGCCCGGGAAGCGCACGAAGGGGGAGGAGATCAAGGGATATACCGGCCCGGATGCCGATCTGGTGGCCCCTTCCATGGAGGAGATGAATGAGCTGATTATTCAGCATGTCGTGATCCGCACCCTGCAGGACATCACCTTGAAGAACGGGCAGCCTGCTTTGAGCATCTTCGACCGGAATACCTGCCATCTGCACATGAACCCCTCAGGGAAATTTATTATTGGAGGCCCGCAGGGGGATGCTGGCCTGACTGGCCGCAAGATCATCATTGATACATACGGTGGCTGGGGCGCCCACGGTGGTGGTGCTTTCTCCGGCAAGGACCCCACCAAGGTGGACCGTTCCGCGGCGTACATCACCCGCCAAATCGCCAAGTCCATCGTGGTCTCCAAGTTGGCAAAGCGAGCATTGGTCCAGTTGTCGTACGCCATTGGCGTTGCCAAGCCCCTGTCCATCTTCGTGGAGACATATGGCTCGGAGCAGGGAGCCCTCTCGGCCGATGACATCACGGACATTGTGAAGCTCGCATTTGACTGCCGTCCCGGTGCCATCGCCAAGTCCCTGCAGCTGAGGGAGCCCAAGTATGTCGAGACAGCAGCCTATTGCCATTTTGGGCGGGAGCCCCGCACGGAGAATGGTATCAAGTATTTTGAGTGGGAGAATCCGGTAGACCTGTCCAAG

> Euglena clara methionine adenosyltransferase (MATX) amino acid translation, partial protein

EFLFSSESVNEGHPDKLCDQVSDSVLDACLTEDPKSKVACETAVKDNMVMVLGEITTQAKLDYDAIVRKAVRNIGFDSFVDDLSSVDSKGLSCDDCEVLVRINKQSPDIAGGVHVGRDEMDLGAGDQGIMFGYASDETQSTMPLTHYLATRLGKTLTDVRKDGSLWWLRPDGKTQVTIKYKQCPDGSVEPLLIHTVLISTQHCEPGKRTKGEEIKGYTGPDADLVAPSMEEMNELIIQHVVIRTLQDITLKNGQPALSIFDRNTCHLHMNPSGKFIIGGPQGDAGLTGRKIIIDTYGGWGAHGGGAFSGKDPTKVDRSAAYITRQIAKSIVVSKLAKRALVQLSYAIGVAKPLSIFVETYGSEQGALSADDITDIVKLAFDCRPGAIAKSLQLREPKYVETAAYCHFGREPRTENGIKYFEWENPVDLSK

>Euglena viridis methionine adenosyltransferase (MATX) mRNA, partial CDS

GAGTTTCTCTTCTCTTCAGAATCTGTGAATGAGGGCCATCCCGATAAGCTGTGCGACCAGGTCTCTGACTCTGTGCTGGATGCCTGCCTTGCCCAAGATCCCAAGAGTAAGGTCGCGTGTGAGACTGCAGTGAAGGACAACATGGTCATGGTTCTGGGTGAGATCACCACCCAAGCTAAATTGGACTATGAGGCCATTGTCCGCAAGGCTGTGCGGAACATCGGATTTGATTCCTTTGTAGACGATCTTTCGAGTGTGGATTCTAAGGGACTGAACTGCGATGACTGTGAAGTACTGGTCCGCATCAACAAGCAGTCCCCCGACATTGCCGGTGGAGTTCACGTTGGTCGTGATGAGCTGGACTTTGGGGCAGGAGATCAGGGCATTATGTTTGGCTATGCAACTGATGAGACCAAGAGCACCATGCCCCTCACCCACTACCTAGCTACCCGCTTGGGTAAGACATTGACTGATGTCCGCAAAGATGGCACCCTTTGGTGGCTGCGCCCTGATGGTAAGACACAGGTGACAATCAAATATAAGCAATGTGCCGATGGGTCTGTGGAACCACTGTTGATCCACACTGTTTTGATCTCTACCCAGCATTGTGAACCTTCCAAGCGAACCAAGGGAGAGGAGATCAAGGGATACAAGGGGGAAGATGCTGGAGAAGTGGCTCCTTCCATGGACCAGATGAATGAGCTCATCATCCAGCATGTGGTTATCCGTACCCTGCAGGACATCACGTTGAAGAACGGACAGCCTGCTCTGAGTATCTTTGACCGTAATACCTGCCATCTTCACATCAACCCCTCCGGCAAGTTCATTATTGGAGGACCTCAGGGTGATGCTGGTCTGACAGGCCGAAAGATTATCATTGACACCTATGGAGGGTGGGGTGCTCATGGAGGTGGTGCTTTCTCTGGAAAGGATCCCACCAAGGTTGATCGATCTGCAGCCTACACCACCCGTCAGATTGCCAAATCTATTGTTGTCTCCAAGCTGGCCAAACGTGCTCTTGTCCAGCTGTCCTATGCCATTGGCGTGGCCAAGCCTCTGTCCATCTTTGTGGAAACCTATGGCTCCGAACAGGGAGCATTGACAGCGGATGACATCACAGACATTGTCAAGCTTGCCTTTGACTGCCGACCAGGTGCCATTGCCAAGTCCCTCCAGCTGACAGAGCCCAAGTATGTGGAGTCTGCTGCCTACTGCCATTTTGGCCGGGAGCCCCGCACCGAGAATGGCATCAAGTTCTTCGAGTGGGAGAACCCTGTCGACCTCTCCTCT

>Euglena viridis methionine adenosyltransferase (MATX) amino acid translation, partial protein

EFLFSSESVNEGHPDKLCDQVSDSVLDACLAQDPKSKVACETAVKDNMVMVLGEITTQAKLDYEAIVRKAVRNIGFDSFVDDLSSVDSKGLNCDDCEVLVRINKQSPDIAGGVHVGRDELDFGAGDQGIMFGYATDETKSTMPLTHYLATRLGKTLTDVRKDGTLWWLRPDGKTQVTIKYKQCADGSVEPLLIHTVLISTQHCEPSKRTKGEEIKGYKGEDAGEVAPSMDQMNELIIQHVVIRTLQDITLKNGQPALSIFDRNTCHLHINPSGKFIIGGPQGDAGLTGRKIIIDTYGGWGAHGGGAFSGKDPTKVDRSAAYTTRQIAKSIVVSKLAKRALVQLSYAIGVAKPLSIFVETYGSEQGALTADDITDIVKLAFDCRPGAIAKSLQLTEPKYVESAAYCHFGREPRTENGIKFFEWENPVDLSS

>Phacus inflexus methionine adenosyltransferase (MATX) mRNA, partial CDS

GAATTCCTTTTCTCATCGGAATCCGTGAATGAAGGCCATCCTGACAAGCTCTGTGACCAGGTTTCAGACTCTGTGCTGGATGCTTGCCTTGCGGTCGACCCGAAGAGCAAGGTGGCTTGTGAATGCGCGGTGAAGGACAACATGGTAATGGTTCTCGGGGAAATCACGACTCAGGCAAAGCTGGATTATGAGAAGATTGTCCGGACTGCCGTGCGCAACATCGGCTTTGATTCCTTTGTTGACGACCTTTCCAGCGTTGACTCGAAGGGGCTGAACTGCGATTCCTGCGAGGTGTTGGTGCGGATCAACAAGCAATCCCCTGATATTGCTGGGGGTGTCCACGTGGGCCGCGATGAGCTCGACTTCGGCGCTGGTGACCAAGGCATCATGTTTGGATATGCGACGGACGAGACCAAGAGCACCATGCCTTTGACGCACTACATGGCGACTCGCCTTGGGAAGACCCTGACCGACGTGCGCAAGAATGGTACGCTTTGGTGGCTGCGCCCTGATGGCAAGACTCAGGTCACCATTAAGTACAAGCAGTGCGCTGATGGCTCTGTTGAGCCCTTAATGATCCACACGGTTCTTATCTCCACCCAGCACTGTGAACCCGGGAAGAGGAAGAAGGGTGAAGAAATTCAGGGCTACACAGGCCCTGATGCCAATGAGGTGGCCCCCAGCATGGAAGAAATGAACGAGCAGATCATCCAGCACGTCATCATCAAGACGCTGCAGGACATCACCCTCAAGAACGGCCAGCCCGCTCTGAGCATTTTCGACCGGAAGACCTGCCATTTGCACATCAATCCCTCTGGCAAGTTCATCATTGGAGGCCCGCAAGGAGATGCCGGTCTGACAGGCCGCAAGATCATCATTGACACCTATGGAGGCTGGGGTGCCCATGGTGGTGGTGCGTTCTCTGGGAAGGATCCGACCAAGGTGGACCGCTCTGCTGCTTACATTACCCGCCAGATTGCCAAGTCCATTGTGGTCTCCAAGTTGGCGAAGCGTGCCCTGGTTCAGTTGTCTTATGCCATTGGTGTTGCCAAGCCCCTTTCCATCTTCGTTGAGACGTATGGCACGGAGCAGGGCGACCTCACTGCCGATGACATCACGGACGTGGTGAAGCTCGCCTTCGATTGCCGCCCTGGCGCGATTGCCAAATCCCTGCAGCTCACCGAGCCTAAGTACGTGGAGACTGCAGCTTATTGCCATTtCGGCCGGGAGCCACGCACCGAGAATGGCATCAAGTATTTTGAGTGGGAGAACCCGAAGGACCTGTCCAAG

>Phacus inflexus methionine adenosyltransferase (MATX) amino acid translation, partial protein

EFLFSSESVNEGHPDKLCDQVSDSVLDACLAVDPKSKVACECAVKDNMVMVLGEITTQAKLDYEKIVRTAVRNIGFDSFVDDLSSVDSKGLNCDSCEVLVRINKQSPDIAGGVHVGRDELDFGAGDQGIMFGYATDETKSTMPLTHYMATRLGKTLTDVRKNGTLWWLRPDGKTQVTIKYKQCADGSVEPLMIHTVLISTQHCEPGKRKKGEEIQGYTGPDANEVAPSMEEMNEQIIQHVIIKTLQDITLKNGQPALSIFDRKTCHLHINPSGKFIIGGPQGDAGLTGRKIIIDTYGGWGAHGGGAFSGKDPTKVDRSAAYITRQIAKSIVVSKLAKRALVQLSYAIGVAKPLSIFVETYGTEQGDLTADDITDVVKLAFDCRPGAIAKSLQLTEPKYVETAAYCHFGREPRTENGIKYFEWENPKDLSK

>Phacus orbicularis methionine adenosyltransferase (MATX) mRNA, partial CDS

GGTCATCCAGACAAGCTCTGTGACCAAGTCTCTGATTCAGTGCTCGATGCTTGCCTGGCAGTGGACCCCAAAAGCAAAGTCGCATGTGAGACGGCTGTGAAAGACAATATGGTGATGGTGCTTGGTGAAATTACCACACAAGCCAAGCTTGATTATGAAAAGATCGTCCGAACAGCAGTCAAAAACATTGGCTTCGATTCGTTTGTGGATGACTTGTCCAGTGTTGATTCCAAGGGGTTGGACCACAAGACATGTGAGGTTCTAGTTCGCATCAACAAGCAGTCCCCTGACATCGCTGGAGGTGTTCACATCGGTCGCGATGAATTGGATTTTGGTGCTGGAGACCAAGGAATTATGTTTGGCTATGCCTCAGATGAGACAAAAAGCACAATGCCCCTGACACATTTTCTTGCCACGCGCCTCGGCAAAACGCTGACAGAAGTTCGCAAGAATGGCACTTTGTGGTGGCTGCGCCCGGACGGAAAGACTCAAGTCACCATTAAGTACAAGCAATGCGCTGATGGTTCTGTGGAGCCACTGATGATTCACACAGTCCTGATTTCCACTCAACACTGTGAGCCAGGCAAAAGGAAAAAAGGCGAAGAGATTCAAGGTTACACGGGAGCAGATGCAGGAGAAATTGCCCCCACCAAGGAGGAAATGGACGAGTTGATGATCCAGCACGTTGTCATCAAGACCTTGCAAGACATCACGTTGAAAAATGGTCAACCTGCCCTGAGCATTTTTGACCGCAACACATGCCATTTGCACATGAACCCCTCAGGCAAATTCATCATTGGTGGTCCTCAGGGAGATGCCGGACTCACTGGCCGTAAAATCATCATTGACACTTACGGTGGCTGGGGCGCTCACGGTGGCGGTGCCTTCTCTGGAAAAGATCCAACCAAGGTTGATCGGTCTGCAGCATACATCACAAGGCAGATTGCCAAGTCCATTGTTGTGTCAAACCTTGCTCGGCGTGCTCTTGTTCAACTGTCATACGCAATTGGGGTGGCGAAGCCCCTTTCAATTTTTGTCGAGACTTATGGATCAGAAAaGGGcGATTTGACTGCTGATGACATCACTGACATCGTGAAGTTGTCTTTCGATTGCCGACCTGGGGCAATTGCAAAGTCTCTTCAGCTCACTGAGCCAAAGTATGTCGAAACAGCAGCTTACTGCCACTTTGGCAGGGACCCTCGCACAGAGAATGGCATCAAGTTCTTCGAGTGGGAGAACCCAGTTGATTTGTCCAAG

>Phacus orbicularis methionine adenosyltransferase (MATX) amino acid translation, partial protein

GHPDKLCDQVSDSVLDACLAVDPKSKVACETAVKDNMVMVLGEITTQAKLDYEKIVRTAVKNIGFDSFVDDLSSVDSKGLDHKTCEVLVRINKQSPDIAGGVHIGRDELDFGAGDQGIMFGYASDETKSTMPLTHFLATRLGKTLTEVRKNGTLWWLRPDGKTQVTIKYKQCADGSVEPLMIHTVLISTQHCEPGKRKKGEEIQGYTGADAGEIAPTKEEMDELMIQHVVIKTLQDITLKNGQPALSIFDRNTCHLHMNPSGKFIIGGPQGDAGLTGRKIIIDTYGGWGAHGGGAFSGKDPTKVDRSAAYITRQIAKSIVVSNLARRALVQLSYAIGVAKPLSIFVETYGSEKGDLTADDITDIVKLSFDCRPGAIAKSLQLTEPKYVETAAYCHFGRDPRTENGIKFFEWENPVDLSK

> Lepocinclis tripteris methionine adenosyltransferase (MATX) mRNA, partial CDS

GAATTTCTTTTTTCCTCGGAATCAGTCAATGAGGGTCACCCTGACAAACTTTGTGACCAAGTGTCGGACTCCGTCTTGGATGCCTGCCTGAAGGAGGATCCCAAGAGCAAGGTGGCATGTGAAACTGCTGTGAAGGACAACATGGTGATGGTGCTGGGAGAAATCACCACGCAAGCTAGGTTGGACTACGAGAAGATCGTGAGGACTGCCGTGCGCAACATTGGTTTCGATTCTTTTGTGGACGACTTGTCCAGCGTGGATTCCAAGGGTTTGAACTGCGACACGTGTGAAGTGCTGGTCCGCATCAACAAGCAGTCGCCCGACATTGCTGGCGGTGTACACGTCGGCAGAGATGACATGGATTTCGGGGCAGGTGACCAAGGAATCATGTTTGGTTATGCCACCGATGAAACCAAGAGTACCATGCCCCTCACCCACTATCTGGCAACCCGCCTTGGCAAAACTTTGACTGAGGTGCGCAAAAATGGAACACTGTGGTGGTTGCGGCCAGATGGGAAAACCCAAGTGACAATCAAGTACAAGCAGTGCAAAGATGGCTCAGTGGAGCCATTGATGATCCACACCGTTCTCATTTCCACTCAGCACTGCGAGCCAGGGAAAAGGAAGAAGGGCGAAGAGATCAAGGGCTACACAGGTGCCGATGCTGGTGAAGTTGCCCCCTCCATGGAACAAATGAATGAGCTCATCATTCAGCACGTCATCATCAAGACCCTGCAGGACATCACCCTGAAGAACGGGCAGCCCGCCCTGACCATCTTCGACCGCAACACCTGCCACCTGCACATCAACCCCTCCGGGAAGTTCATCATCGGCGGGCCGCAGGGGGACGCCGGGCTCACCGGCCGCAAGATCATCATCGACACCTACGGCGGGTGGGGCGCGCACGGTGGGGGGGCCTTCTCCGGAAAGGACCCCACCAAGGTGGACCGCTCCGCCGCGTACATCACCCGCCAGATGGCCAAGTCCATCGTCGTCAGCGGGCTGGCGAAGCGAGCCCTGGTGCAGCTGTCGTATGCGATTGGCGTTGCCAAGCCCCTGTCCATTTTTGTGGAAACCTACGGTTCGGAGCAGGGGGCATTGACAGCTGCGGACATCACGGACATTGTGAAGCTCGCCTTCGACTGCCGGCCGGGCGCCATCGCCAAGTCCCTGCAGCTGACGGAGCCCAAGTACGTGGAGACCGCGGCATACTGCCACTTCGGCAGGGAGCCGCGCACCGAGAATGGCATCAAGTTCTTTGAATGGGAGAACCCCGTTGACCTGTCCAAG

>Lepocinclis tripteris methionine adenosyltransferase (MATX) amino acid translation, partial protein

EFLFSSESVNEGHPDKLCDQVSDSVLDACLKEDPKSKVACETAVKDNMVMVLGEITTQARLDYEKIVRTAVRNIGFDSFVDDLSSVDSKGLNCDTCEVLVRINKQSPDIAGGVHVGRDDMDFGAGDQGIMFGYATDETKSTMPLTHYLATRLGKTLTEVRKNGTLWWLRPDGKTQVTIKYKQCKDGSVEPLMIHTVLISTQHCEPGKRKKGEEIKGYTGADAGEVAPSMEQMNELIIQHVIIKTLQDITLKNGQPALTIFDRNTCHLHINPSGKFIIGGPQGDAGLTGRKIIIDTYGGWGAHGGGAFSGKDPTKVDRSAAYITRQMAKSIVVSGLAKRALVQLSYAIGVAKPLSIFVETYGSEQGALTAADITDIVKLAFDCRPGAIAKSLQLTEPKYVETAAYCHFGREPRTENGIKFFEWENPVDLSK

>Trachelomonas ellipsoidalis methionine adenosyltransferase (MATX) mRNA, partial CDS

GAATTCCTGTTTTCTTCTGAATCAGTCAATGAAGGTCACCCTGATAAGTTGTGTGATCAAGTGTCCGACTCTGTTTTGGATGCCTGTTTGGCAGTTGATCCAAAGAGCAAGGTAGCTTGTGAAACTGCAGTGAAGGACAACATGGTCATGGTTCTTGGGGAAATCACCACCCAGGCAAAGTTGGATTACGAAAAGATTGTCCGGACAGCAGTCAGAAACATTGGCTTTGATTCATTTGTTGATGACTTGTCCAGCGTTGATTCCAAGGGGTTAAATTGTGATGACTGTGAAGTTCTTGTGCGGATTAACAAGCAATCTCCTGACATTGCTGGAGGAGTGCACGTTGGGAGGGATGACATGGATGTTGGAGCTGGCGATCAAGGAATCATGTTTGGATATGCCACAGATGAAACTCAGAGCACCATGCCACTGACACATTACCTTGCAACTCGCCTTGGGAAAACCCTCACGGAAGTGCGAAAGGATGGTTCTCTTTGGTGGTTGCGTCCCGATGGAAAGACACAAGTGACAATCAAGTACAAGCAATGTGCAGATGGTTCTGTGGAGCCCCTGTCGATACACACAGTGCTTATTTCCACGCAGCACGCTGAACCTGGCAAGAGAAAGAAGGGGGAGGAGATACGGGGTTACAAAGGGCCAGATGCTGATGAAGTGGCACCAAGCATGGAACAAATGAATGAATTGATCATTCAACATGTGGTCATTCGGACTCTGGAAAACATTACTTTGAAGAATGGGCAGCCAGCTTTGAGCCTCTTTGATCGCAACTCATGCCACCTTCACATCAATCCTTCTGGCAAGTTCATCATTGGAGGCCCACAAGGTGATGCTGGGTTGACAGGGCGGAAGATCATCATTGACACATATGGTGGTTGGGGTGCCCATGGTGGTGGTGCCTTTTCTGGGAAGGACCCCACCAAGGTTGACCGGTCTGCTGCATACATCACACGCCAGATTGCCAAGTCCATCGTTGTTTCCAAGCTTGCCAAACGTGCTTTGGTGCAGCTGTCATATGCCATCGGGGTTGCCAAGCCTCTCTCTATCTTTGTGGAGACCTATGGGTCAGAGCAGGGGGCCCTGACAGCAAGTGACATCACAGATATTGTGAAGCTTGCATTCGATTGCCGCCCTGGCGCCATTGCCAAGTCATTGCAGCTGACAGAGCCCAAATATGTGGAGACTGCTGCGTATTGCCATTTTGGGAGGGAGCCCCGCACTGAGAATGGCATCAAGTATTTTGAGTGGGAGAACCCAGTGGACCTCTCCAAA

>Trachelomonas ellipsoidalis methionine adenosyltransferase (MATX) amino acid translation, partial protein

EFLFSSESVNEGHPDKLCDQVSDSVLDACLAVDPKSKVACETAVKDNMVMVLGEITTQAKLDYEKIVRTAVRNIGFDSFVDDLSSVDSKGLNCDDCEVLVRINKQSPDIAGGVHVGRDDMDVGAGDQGIMFGYATDETQSTMPLTHYLATRLGKTLTEVRKDGSLWWLRPDGKTQVTIKYKQCADGSVEPLSIHTVLISTQHAEPGKRKKGEEIRGYKGPDADEVAPSMEQMNELIIQHVVIRTLENITLKNGQPALSLFDRNSCHLHINPSGKFIIGGPQGDAGLTGRKIIIDTYGGWGAHGGGAFSGKDPTKVDRSAAYITRQIAKSIVVSKLAKRALVQLSYAIGVAKPLSIFVETYGSEQGALTASDITDIVKLAFDCRPGAIAKSLQLTEPKYVETAAYCHFGREPRTENGIKYFEWENPVDLSK

>Euglena stellata methionine adenosyltransferase (MATX) mRNA, partial CDS

GAGTTCCTTTTCTCTTCCGAATCTGTTAATGAAGGTCATCCTGACAAGCTTTGCGACCAGGTTTCAGACTCCGTATTGGATGCTTGCCTCGCTCAAGATCCCAAGAGTAAAGTTGCTTGTGAAACAGCCGTGAAGGATAATATGGTGATGGTTTTGGGAGAAATTACAACCCAAGCCAAGCTGGACTATGAGGCAATTGTCCGCCAGGCCGTCCGAAATATCGGCTTTGATTCCTTTGTTGACGACCTTGCCAGTGTTGATTCCAAGGGGCTGAACTGTGATGATTGTGAAGTGCTTGTCCGTATCAACAAGCAATCCCCAGATATTGCTGGTGGAGTGCACGTTGGTAAGGATGAGATGGATGTGGGTGCTGGAGATCAGGGAATTATGTTTGGATATGCTTCTGATGAGACCAAGAGCACCATGCCCTTGACTCACTACATGGCTACCCGACTGGGCAAAACGCTCACTGAAGTTCGCAAGAATGGCACCCTCTGGTGGTTGCGTCCTGATGGCAAGACCCAGGTGACCATCAAGTACAAGCAATGCGCTGATGGGTCAGTTGAGCCTCTGATGATCCACACCGTGCTGATCTCCACCCAGCACTCTGAACCCGGCAAGCGGACCAAGGGAGAGGAAATCCGCGGGTACAAGGGTGCCGATGCCGGTGAGGTAGCCCCAAGCATGGAGCAGATGAACGAGCTCATCATCCAGCACGTTGTCGTTAAAACCTTGGAGGAGATCACTCTGAAGAACGGTCAATCCGCTCTGAGCATCTTCGACCGCAACACTTGCCATCTGCACATCAACCCCTCTGGCCGGTTCATCATTGGTGGACCACAGGGAGACGCTGGTCTGACTGGCCGCAAGATCATCATTGACACCTATGGCGGCTGGGGAGCACATGGTGGTGGAGCTTTCTCTGGCAAGGACCCGACCAAGGTTGACCGGTCGGCCGCTTACATCACCCGCCAGATCGCTAAGTCCATCGTAGTCTCCGGCCTGGCCAAGCGAGTGCTGGTGCAGCTCTCGTACGCCATCGCCGTTGCTAAGCCTTTGTCCGTCTTTGTTGAGACCTATGGCTCAGAGCAGGGCGCTCTGACCGCCGATGACATCACGGATATCGTGAAGCTGAACTTCGACTGCCGTCCCGGAGCGATCGCTAAATCACTGCAGCTGCGAGAGCCCAAGTACACCGAATCCGCCGCCTACTGCCACTTTGGGCGGGAGCCACGCACCGAGAACGGCATCAAGTATTTTGAGTGGGAGAACCCCGTTGATCTGTCCAAG

>Euglena stellata methionine adenosyltransferase (MATX) amino acid translation, partial protein

EFLFSSESVNEGHPDKLCDQVSDSVLDACLAQDPKSKVACETAVKDNMVMVLGEITTQAKLDYEAIVRQAVRNIGFDSFVDDLASVDSKGLNCDDCEVLVRINKQSPDIAGGVHVGKDEMDVGAGDQGIMFGYASDETKSTMPLTHYMATRLGKTLTEVRKNGTLWWLRPDGKTQVTIKYKQCADGSVEPLMIHTVLISTQHSEPGKRTKGEEIRGYKGADAGEVAPSMEQMNELIIQHVVVKTLEEITLKNGQSALSIFDRNTCHLHINPSGRFIIGGPQGDAGLTGRKIIIDTYGGWGAHGGGAFSGKDPTKVDRSAAYITRQIAKSIVVSGLAKRVLVQLSYAIAVAKPLSVFVETYGSEQGALTADDITDIVKLNFDCRPGAIAKSLQLREPKYTESAAYCHFGREPRTENGIKYFEWENPVDLSK

> Euglena hiemalis methionine adenosyltransferase (MATX) mRNA, partial CDS

GAATTTCTCTTCTCCTCTGAGTCAGTCAATGAGGGTCATCCGGATAAGCTGTGCGACCAGGTTTCAGACTCTGTTCTGGATGCCTGCTTGGCTCAGGACCCACTCAGCAAAGTGGCTTGTGAGGCAGCTGTGAAGGATAATATGGTCATGGTTCTGGGTGAAATCACCACTCAAGCCAAGCTGGACTACGATGCCATTGTGCGCAATGCTGTCCGCAACATCGGCTTTGATGCATTTGTTGATGACTTGAGCAGTGTGGACTCCAAGGGGTTGAACTGTGATGATTGTGAGGTCCTTGTCCGGATCAACAAGCAGTCCCCAGACATTGCTGGCGGTGTGCACGTTGGTCGTGATGAGATGGATGTTGGAGCTGGAGATCAGGGCATTATGTTTGGCTATGCCACTGATGAAACTGAGAGCACCATGCCCTTGACACACTACGTTGCTACTCGCCTTGGCAAGACGCTGACCGAGGTGCGCAAGGATGGAAGTTTGTGGTGGTTGCGTCCTGATGGCAAGACGCAGGTGACCATCAAGTACAAGCAGTGCGCGGATGGAGCCGTGGAGCCACTAATGATCCACACCATCCTGATCTCCACCCAGCACGTGGAGCCGGGCAAGCGCAAGAAGGGGGAGGAGCTCAAGGGGTACAAGGGGCCCGACGCGGAGGAGGTGGCCCCAAGCATGGAGCAGATGAACGAGCTGATGATCCAGCACGTCGTGATCCGCACCCTGGAGAACATCACGCTGAAGAACGGGAAGCCGGCCATCAGCATCTTCGACCGCAGCACGTGCCACCTGCACATGAACCCGTCAGGGAAGTTCATCATCGGCGGCCCGCAGGGGGACGCCGGCCTCACCGGCCGCAAGATCATCATTGACACCTACGGCGGGTGGGGCGCTCACGGGGGCGGGGCCTTCTCTGGCAAGGACCCCACCAAGGTGGACCGCTCCGCGGCCTACATCACCCGCCAGATCGCCAAGTCCATCGTCGTCTCCAAGCTGGCGCGCCGGGCACTGGTGCAGCTGTCCTACGCCATCGCCGTCGCCAAGCCCCTGTCCGTGTTCGTGGAGACGTACGGGTCGGAGCAGGGCGCCCTGACCGCCGACGACATCACCGACATCGTCAAGCTGAACTTCGACTGCCGCCCCGGTGCCATCGCCAAGTCTCTGCAGCTGCGGGAACCGAAGTACGTGGAGTCTGCGGCCTACTGCCACTTCGGACGGGAGCCCCGCACCGAGAACGGCATCAAGTATTTTGAGTGGGAGAACCCGGTGGACCTCTCCAAG

>Euglena hiemalis methionine adenosyltransferase (MATX) amino acid translation, partial protein

EFLFSSESVNEGHPDKLCDQVSDSVLDACLAQDPLSKVACEAAVKDNMVMVLGEITTQAKLDYDAIVRNAVRNIGFDAFVDDLSSVDSKGLNCDDCEVLVRINKQSPDIAGGVHVGRDEMDVGAGDQGIMFGYATDETESTMPLTHYVATRLGKTLTEVRKDGSLWWLRPDGKTQVTIKYKQCADGAVEPLMIHTILISTQHVEPGKRKKGEELKGYKGPDAEEVAPSMEQMNELMIQHVVIRTLENITLKNGKPAISIFDRSTCHLHMNPSGKFIIGGPQGDAGLTGRKIIIDTYGGWGAHGGGAFSGKDPTKVDRSAAYITRQIAKSIVVSKLARRALVQLSYAIAVAKPLSVFVETYGSEQGALTADDITDIVKLNFDCRPGAIAKSLQLREPKYVESAAYCHFGREPRTENGIKYFEWENPVDLSK

> Lepocinclis playfairiana methionine adenosyltransferase (MATX) mRNA, partial CDS

GAATTTTTATTTTCTTCCGAGTCTGTGAACGAAGGCCATCCCGACAAGCTTTGCGACCAAGTATCCGATTCAGTTCTTGATGCTTGCTTGCGTGAGGATCCACTGAGCAAAGTGGCCTGTGAGACTGCTGTGAAGGACAATATGGTAATGGTGCTTGGAGAAATTACAACCAACGCTAAATTGGACTATGAAAAGATTGTCCGCGATGCTGTCCGTAACATTGGCTTTGATTCCTTCGTGGACGATCTGTCAAGTGTGGATTCGAAGGGTTTGAATTGTGAAGACTGTGAAGTTTTGGTTCGCATAAACAAGCAATCTCCTGACATTGCAGGGGGTGTTCATGTTGGCCGAGATGAAATGGATGTTGGTGCTGGGGATCAGGGAATTATGTTTGGATATGCCTCAGATGAAACCAAGAGCACCATGCCTCTTACTCATTACTTGGCGACAAGGCTTGGGAAGACATTGACTGAAGTAAGGAAAAGTGGGCAGCTATGGTGGCTGCGCCCCGATGGGAAGACTCAGGTCACCATTAAGTACAAGCAAAATGCAGATGGCTCAGTTGAACCCCTGCTGATCCACACAGTCCTAATTTCCACGCAGCACTGTGAACCTGGAAAGAGGAAGAAGGGTGAAGAAATTAAGGGTTACAAGGGAGCTGATGCTGACGATGTGGCTCCTTCGATGGAACAAATGAATGAGCTTATCATCGAGCACGTGATCATCAAAACCCTTCAAGACATCACACTGAAGAATGGAAAACCTGCTTTATCAATCTTTGACCGCAGCACTTGCCACTTGCATATCAATCCTTCTGGGAAGTTCATCATTGGCGGACCCCAGGGAGATGCTGGTCTGACCGGAAGGAAAATCATCATTGATACCTATGGAGGCTGGGGAGCTCATGGCGGTGGAGCATTCTCTGGGAAAGATCCCACCAAAGTGGACCGATCTGCCGCATACATTACCCGCCAAATCGCCAAGTCTGTCGTTGCCAGTGGACTTGCACGCCGAGCACTGGTTCAATTATCTTATGCCATTGGGGTTGCTAAACCTTTATCCATCTTTGTGGAGACCTATGGCTCGGAGCAGGGCAAATTGACTGCTGATGACATCACTGACATTGTTAAGCTTGAATTTGACTGCCGTCCTGGGGCCATTGCGAAGTCTTTGCAGCTCAGGGAACCCAAGTACGTTGAGACTGCTGCATACTGCCACTTTGGGCGAGAGCCCCGTACAGAGAATGGCATCAAGTACTTCGCTTGGGAGAACCCTGTTGACTTGTCCAAA

>Lepocinclis playfairiana methionine adenosyltransferase (MATX) amino acid translation, partial protein

EFLFSSESVNEGHPDKLCDQVSDSVLDACLREDPLSKVACETAVKDNMVMVLGEITTNAKLDYEKIVRDAVRNIGFDSFVDDLSSVDSKGLNCEDCEVLVRINKQSPDIAGGVHVGRDEMDVGAGDQGIMFGYASDETKSTMPLTHYLATRLGKTLTEVRKSGQLWWLRPDGKTQVTIKYKQNADGSVEPLLIHTVLISTQHCEPGKRKKGEEIKGYKGADADDVAPSMEQMNELIIEHVIIKTLQDITLKNGKPALSIFDRSTCHLHINPSGKFIIGGPQGDAGLTGRKIIIDTYGGWGAHGGGAFSGKDPTKVDRSAAYITRQIAKSVVASGLARRALVQLSYAIGVAKPLSIFVETYGSEQGKLTADDITDIVKLEFDCRPGAIAKSLQLREPKYVETAAYCHFGREPRTENGIKYFAWENPVDLSK

>Euglenaria anabaena methionine adenosyltransferase (MATX) mRNA, partial CDS

GTCAATGAGGGCCACCCAGACAAGCTGTGCGATCAGGTGTCGGACTCCGTGCTGGATGCCTGCCTGGCTCAGGACCCCAAGAGCAAGGTGGCCTGTGAAACGGCTGTGAAGGACAACATGGTCATGGTACTCGGGGAGATCACCACACAGGCCAAGCTTGACTACGAGAAGATCGTGCGTACAGCAGTGCGTAACATCGGCTTCGACTCGTTCATTGACGACCTTACCAGTGTGGAGTCGAAGGGGCTGGACTGCGACAACTGCGAGGTGCTGGTGCGGATCAACAAGCAGTCCCCCGACATTGCGGGAGGCGTGCATGTTGGTCGCGACGATATGGACGTGGGCGCTGGCGATCAGGGCATTATGTTCGGCTACGCCACAGACGAGACGCAGAGCACGATGCCCCTGACGCACTACATGGCTACCAGGCTGGGCAAGACCTTGACGGAAGTTCGCAAGGACGGCACCCTGTGGTGGCTGCGCCCCGACGGCAAGACGCAGGTGACCATCAAGTACCGGCAGTGCGCGGATGGGTCGGTGGAGCCGCTGCTGATCCACACAGTGCTCATCTCTACCCAGCACTGTGAGCCCGCGAAGCGCACGAAGGGGGAGGAGATCAAGGGCTACAAGGGGTCCGATGCCGACGAGGTTGCCCCGTCGATGGAGGACATGAACGAGCTGATCGTGCAGCACGTCGTGATCCGCACCCTGCAGAACATCACCCTGAAGAACGGAAAGCCTGCGCTGTCCATCTTCGACCGGAACACCTGCCATTTGCACATCAACCCGTCTGGGAAGTTCATCATCGGCGGGCCCCAGGGCGACGCGGGGCTGACGGGCCGGAAGATCATCATCGACACCTACGGCGGGTGGGGCGCCCACGGCGGCGGGGCGTTCTCCGGGAAGGACCCCACCAAGGTGGACCGCTCCGCGGCGTACATCACGCGGCAGATCGCGAAGTCCATCGTGGTGTCCAATTTGGCGCGGCGTGCTCTGGTGCAGCTGTCCTACGCCATCGGGGTGGCCAAGCCCCTCTCCGTCTTCGTGGAGACCTACGGGTCGGAGCAGGGCGACCTAACGGCGGCGGACATCACGGACATCGTGAAGCTTGCATTCGACTGTCGCCCGGGGGCTATCGGGAAGTCACTGCAGCTGAGGGAGCCGAAGTACACGGAGACGGCGGCGTACTGCCACTTCGGGCGGGAGCCCCGCGTTGAGGGCACCATCAAGTTCTTCGAGTGGGAGCGGCCGGTGGACCTATCCAAG

>Euglenaria anabaena methionine adenosyltransferase (MATX) amino acid translation, partial protein

VNEGHPDKLCDQVSDSVLDACLAQDPKSKVACETAVKDNMVMVLGEITTQAKLDYEKIVRTAVRNIGFDSFIDDLTSVESKGLDCDNCEVLVRINKQSPDIAGGVHVGRDDMDVGAGDQGIMFGYATDETQSTMPLTHYMATRLGKTLTEVRKDGTLWWLRPDGKTQVTIKYRQCADGSVEPLLIHTVLISTQHCEPAKRTKGEEIKGYKGSDADEVAPSMEDMNELIVQHVVIRTLQNITLKNGKPALSIFDRNTCHLHINPSGKFIIGGPQGDAGLTGRKIIIDTYGGWGAHGGGAFSGKDPTKVDRSAAYITRQIAKSIVVSNLARRALVQLSYAIGVAKPLSVFVETYGSEQGDLTAADITDIVKLAFDCRPGAIGKSLQLREPKYTETAAYCHFGREPRVEGTIKFFEWERPVDLSK

>Monomorphina parapyrum methionine adenosyltransferase (MATX) mRNA, partial CDS

GAGTTCCTCTTCTCCTCCGAGTCAGTCAATGAGGGTCACCCAGACAAACTGTGCGATCAGGTCTCGGACTCAGTGCTTGATGCCTGCCTCAAGGTGGATCCCAAAAGCAAGGTGGCCTGTGAGACAGCTGTGAAGGACAACATGGTGATGGTGCTCGGCGAGATCACCACCCAGGCGAAGCTGGACTATGAGGCCATCGTGCGCACCGCTGTCCGCAACATTGGATTCGACTCGTTCGTGGACGACCTGAGCAGCGTGGATTCCAAGGGACTGAACTGTGATGATTGTGAAGTGTTGGTGCGCATCAACAAGCAGTCGCCCGACATTGCAGGCGGGGTGCACGTGGGGCGCGATGACATGGACTTTGGAGCGGGCGACCAGGGCATCATGTTCGGGTACGCCACGGACGAGACTCAGAGCACGATGCCGCTGACGCACTACCTTGCCACGCGCCTCGGGCGCACGCTGACGGAGGTGCGCAAGAACGGCACGCTGTGGTGGCTGCGCCCCGACGGGAAGACGCAGGTGACAATCAAGTACAGGCAGTGCGCCGACGGCTCCGTGGAGCCGCTGATGATCCACACCGTGCTGATCTCCACACAGCACTGCGAGCCCGGCAAGCGCACGAAGGGCGCGGAGATCAAGGGCTACAAGGGCGCGGACGCCGGCGAGGTGGCCCCGTCGATGGAGCAGATGAACGAGCTGATCATCCAGCACGTTGTGATCCGCACCCTGCAGGACATCACGTTGAAGAACGGCAAGCCGGCGCTGAGCATCTTCGACCGCAAGACGTGCCACCTGCACATCAACCCCTCCGGCAAGTTCATCATCGGCGGCCCCCAGGGCGACGCCGGGCTGACCGGCCGCAAGATCATCATCGACACCTATGGTGGCTGGGGTGCGCACGGTGGTGGCGCCTTCTCTGGCAAGGACCCCACCAAGGTGGACCGCTCCGCGGCATACATCACGCGCCAGATCGCCAAGTCCATCGTCGTCTCGCGCCTCGCGAAGCGCGCGCTGGTGCAGCTGTCGTACGCCATCGGTGTTGCCAAGCCGCTCTCCATCTTCGTCGAGACGTACGGGTCGGAGATGGGCGAGCTGACGGCCGATGACATCACGGACATCGTGAAGCTGGCGTTCGACTGCCGCCCCGGCGCGATCGCCAAGTCGCTGCAGCTGACCGAGCCCAAGTACACGGAGACGGCGGCGTACTGCCACTTCGGGCGCGAGCCGCGCACCGAGAACGGCATCAAGTTCTTCGAGTGGGAGAACCCCGTGGACCTGTCCGCC

>Monomorphina parapyrum methionine adenosyltransferase (MATX) amino acid translation, partial protein

EFLFSSESVNEGHPDKLCDQVSDSVLDACLKVDPKSKVACETAVKDNMVMVLGEITTQAKLDYEAIVRTAVRNIGFDSFVDDLSSVDSKGLNCDDCEVLVRINKQSPDIAGGVHVGRDDMDFGAGDQGIMFGYATDETQSTMPLTHYLATRLGRTLTEVRKNGTLWWLRPDGKTQVTIKYRQCADGSVEPLMIHTVLISTQHCEPGKRTKGAEIKGYKGADAGEVAPSMEQMNELIIQHVVIRTLQDITLKNGKPALSIFDRKTCHLHINPSGKFIIGGPQGDAGLTGRKIIIDTYGGWGAHGGGAFSGKDPTKVDRSAAYITRQIAKSIVVSRLAKRALVQLSYAIGVAKPLSIFVETYGSEMGELTADDITDIVKLAFDCRPGAIAKSLQLTEPKYTETAAYCHFGREPRTENGIKFFEWENPVDLSA

> Eutreptiella braarudii methionine adenosyltransferase (MATX) mRNA, partial CDS

GAGTTCCTTTTCTCCTCTGAGTCTGTGAATGAGGGTCACCCTGATAAGCTGTGCGACCAGGTTTCCGACTCCGTTCTGGATGCCTGCTTGACCGCTGACCCCCTGAGCAAGGTGGCCTGCGAGACTGCCGTGAAGGACAACATGGTCATGGTTCTTGGTGAGATCACCACCGGCGCCAAGTTGGACTACGAGGCCATTGTTCGCAAGGCTTGCCGCCACATCGGTTTCGACTCCTATGTGGATGACTTGTCCAGTGTGGAGTCCAAGGGTTTGAACTGCGATGACTGCGAGGTGCTTGTCCGTATCAACGCTCAGTCTCCCGACATTGCTGGCGGTGTGCACGTTGGCCGTGACGAGATGGACGTTGGTGCTGGTGACCAGGGTATTATGTTCGGATATGCCTCTGACGAGACCAAGAGCACCATGCCTTTGACTCACTATCTTGCCACCAAGCTGGGCAAGACCCTCACTGACGTTCGCAAGAACGGCAAGTTGTGGTGGTTGCGACCTGACGGTAAGACTCAGGTGACCATCAAGTACAAGCAGCACGCTGACGGTTCCGTGGAGCCTCTTATGATTCACACCGTGCTGATCTCCACCCAGCACGCCGAGCCTCTGAAGAGGACCAAGGGTGCCGAGATCGCTGGCTACAAGGGTGCGGACGCCGGTGAGGTGGCCCCTTCCATGGAGGACATGAACAAGCAGATCCTTGAGCAGGTCATCAAGGCAACCCTTCGTGACATCACGTTGAAGAACGGCAAGCCTGCCATCAGCATCTTTGACGAGAAGACCTGCCACACCCACATCAACCCATCTGGCAAGTTCATCATCGGTGGTCCTCAGGGCGATGCCGGTCTTACTGGCCGTAAGATCATCATCGACACCTATGGTGGTTGGGGTGCCCACGGTGGTGGTGCTTTCTCCGGCAAGGACCCCACCAAGGTGGACCGCTCTGCTGCCTACATCACCCGCCAGATGGCTAAGTCCATCGTGACTTCCGGACTTGCCAAGCGCGCCCTGGTGCAGCTTTCCTACGCCATTGGTGTGGCCAAGCCTCTGTCCGTGTTCGTCGAGACCTACGGCTCCGAGCAGGGTGCCTTGACTGCTGATGATATCACTGACGTCATCAAGCTTGCCTTCGACTGCCGCCCTGGTGCCATCGCCAAGTCCTTGGCCCTGCGTGAGCCCAAGTACGTGGAGAGTGCAGCTTACTGCCATTTCGGCCGTGAGCCTCGCACCGAGAACGGCATCAAGTACTTCGAGTGGGAGAACCCTGTGGATCTCTCCAAG

>Eutreptiella braarudii methionine adenosyltransferase (MATX) amino acid translation, partial protein

EFLFSSESVNEGHPDKLCDQVSDSVLDACLTADPLSKVACETAVKDNMVMVLGEITTGAKLDYEAIVRKACRHIGFDSYVDDLSSVESKGLNCDDCEVLVRINAQSPDIAGGVHVGRDEMDVGAGDQGIMFGYASDETKSTMPLTHYLATKLGKTLTDVRKNGKLWWLRPDGKTQVTIKYKQHADGSVEPLMIHTVLISTQHAEPLKRTKGAEIAGYKGADAGEVAPSMEDMNKQILEQVIKATLRDITLKNGKPAISIFDEKTCHTHINPSGKFIIGGPQGDAGLTGRKIIIDTYGGWGAHGGGAFSGKDPTKVDRSAAYITRQMAKSIVTSGLAKRALVQLSYAIGVAKPLSVFVETYGSEQGALTADDITDVIKLAFDCRPGAIAKSLALREPKYVESAAYCHFGREPRTENGIKYFEWENPVDLSK

>Trachelomonas volvocina methionine adenosyltransferase (MATX) mRNA, partial CDS

GAATTTTTGTTTTCCTCTGAGTCAGTCAATGAGGGACATCCTGACAAGCTGTGTGATCAAGTTTCAGACTCTGTGCTGGATGCTTGTCTTGCAGCTGACCCCAAAAGCAAGGTGGCATGTGAAACAGCCGTGAAGGATAACATGGTGATGGTGTTGGGTGAGATCACCACCCAGGCCAAGCTGGACTATGAAAAGATTGTCCGCAGTGCAGTCCGTAACATCGGATTCGATTCCTTTGTGGATGATTTGTCCAGCGTGGATTCCAAGGGGCTGAACTGCGATGATTGTGAAGTGTTGGTCCGCATCAACAAGCAGTCTCCGGACATTGCTGGGGGCGTGCATGTTGGACGAGATGACATGGATGTGGGTGCTGGGGACCAGGGTATCATGTTTGGCTATGCCTCTGATGAGACTCAAAGCACCATGCCCCTCACCCACTACTTGGCAACCCGCTTGGGCAAGACCCTGACCGACGTTCGCAAGAATGGCACCTTGTGGTGGTTGCGCCCCGATGGCAAGACCCAGGTCACAATCAAGTATCGCCAGTGTGCTGATGGTGCTGTTGAACCCTTGATGATCCACACAGTGCTGATCTCCACCCAGCATGCCGAGCCAGGGAAGCGCACGAAGGGGGAGGAGATCCGTGGCTACAAGGGGGCAGATGCAAACGAAGTGGCCCCATCGATGGACCAGATGAATGAATTGATCATCCAGCACGTCGTCATTCGTACATTGGAGGACATCAAGCTGAAGAACGGCAAGCCAGCCCTCAGCATCTTCGACCGCAAGACATGCCATTTGCACATCAACCCATCGGGCAAGTTCATTATCGGAGGCCCACAGGGAGACGCCGGGTTGACGGGACGCAAGATCATCATCGACACCTATGGCGGATGGGGTGCGCATGGTGGTGGTGCATTCTCCGGAAAGGACCCCACAAAGGTCGACCGATCAGCAGCATACATCACACGCCAGATCGCAAAGTCCATCGTCGTTTCCAAGCTTGCCCGCCGGGCCCTCGTTCAGCTGTCCTATGCCATCGGTGTGGCAAAACCACTGTCCGTCTTTGTGGAGACATACGGATCGGAGCAAGGCAGCTTGACGGCTGATGACATCACCGATATCGTGAAGTTGGCGTTTGACTGCCGCCCCGGCGCCATCGCCAAGTCCCTGCAACTGACCGAGCCAAAGTATGTGGAGACCGCGGCGTACTGCCATTTCGGGCGGGAACCACGGACACAGAACGGCATCAAGTTCTTCGAATGGGAGAACCCCGTTGATTTGTCCAAG

>Trachelomonas volvocina methionine adenosyltransferase (MATX) amino acid translation, partial protein

EFLFSSESVNEGHPDKLCDQVSDSVLDACLAADPKSKVACETAVKDNMVMVLGEITTQAKLDYEKIVRSAVRNIGFDSFVDDLSSVDSKGLNCDDCEVLVRINKQSPDIAGGVHVGRDDMDVGAGDQGIMFGYASDETQSTMPLTHYLATRLGKTLTDVRKNGTLWWLRPDGKTQVTIKYRQCADGAVEPLMIHTVLISTQHAEPGKRTKGEEIRGYKGADANEVAPSMDQMNELIIQHVVIRTLEDIKLKNGKPALSIFDRKTCHLHINPSGKFIIGGPQGDAGLTGRKIIIDTYGGWGAHGGGAFSGKDPTKVDRSAAYITRQIAKSIVVSKLARRALVQLSYAIGVAKPLSVFVETYGSEQGSLTADDITDIVKLAFDCRPGAIAKSLQLTEPKYVETAAYCHFGREPRTQNGIKFFEWENPVDLSK

>Eutreptia viridis methionine adenosyltransferase (MATX) mRNA, partial CDS

GAGTTCTTGTTCTCCTCTGAATCTGTTAACGAGGGTCATCCTGATAAGCTCTGCGACCAAGTGTCCGACTCGGTTCTAGATGCCTGCCTGAAAGAGGATCCTCTCAGCAAGGTTGCCTGTGAAACCGCTGTCAAGGACAACATGGTGATGGTCTTGGGTGAAATCACCACCAAGGCCAAGTTGGACTACGAAGCGATTGTGCGCAAGGCAGTCCGCAACATCGGTTTCGACTCGTTTGTTGACGACCTTGCCAGTGTCGACTCGAAGGGTCTGAACTGTGACGATTGCGAGGTTCTTGTTCGCATCAACAAGCAGTCTCCTGATATTGCAGGTGGTGTGCACGTTGGTCGCGACGAGATGGATGTTGGAGCCGGGGATCAAGGTATTATGTTTGGTTATGCCACTGACGAAACCAAGAGCACGATGCCCTTGACCCACTACCTTGCCACGAAACTGGGAAAGACTCTCACCGACGTTCGGAAGAGTGGTGAGCTTTGGTGGTTGCGCCCTGATGGCAAGACCCAGGTTACCATCAAGTACAAACAATGTGCCGACGGCTCGGTAGAGCCTCTGATGATCCACACGATCTTGATCTCCACTCAGCATGCCGAGCCTATGAAGAGGACCAAGGGTGCCGAAATAGCCGGTTACAAGGGTGCTGACTCTGGAGAAACCGCTCCTTCAATGGAGAAGATGAACGAACTCATTGTAGAGTATGTAGTTAAGAGGACGCTGAAGGAGATCACGTTGAAGAACGGCGAGCCTGCCCTCAGCATCTTCGATGAGAAGACTTGCCACTTGCACATCAACCCATCTGGCAAGTTCATCATTGGTGGTCCACAAGGTGATGCCGGTCTCACTGGTCGCAAGATCATCATTGACACCTATGGAGGCTGGGGTGCCCATGGAGGTGGTGCCTTCTCTGGCAAGGATCCCACCAAGGTTGATCGTTCCGCTGCCTACATCACCCGTCAAATGGCAAAGTCTATTGTGACCTCTGGACTTGCAAAACGTGCTCTCGTGCAGCTTTCATATGCCATTGGTGTTGCAAAGCCGCTTTCCATTTTTGTTGAGACCTACGGGTCCGAGCTCGGCGAGTTGACAGCTGACGACATCACAGACGTCATCAAGCTTGCATATGACTGCCGGCCAGGTGCCATTGCTAAACATTTGCAGCTGACTGAACCCAAGTACGTCGAGACAGCAGCTTACTGCCACTTTGGACGAGAGCCTCGTACTGAAAATGGCATCAAATATTTTGAGTGGGAGAATCCAGTTGACCTTTCGAAG

>Eutreptia viridis methionine adenosyltransferase (MATX) amino acid translation, partial protein

EFLFSSESVNEGHPDKLCDQVSDSVLDACLKEDPLSKVACETAVKDNMVMVLGEITTKAKLDYEAIVRKAVRNIGFDSFVDDLASVDSKGLNCDDCEVLVRINKQSPDIAGGVHVGRDEMDVGAGDQGIMFGYATDETKSTMPLTHYLATKLGKTLTDVRKSGELWWLRPDGKTQVTIKYKQCADGSVEPLMIHTILISTQHAEPMKRTKGAEIAGYKGADSGETAPSMEKMNELIVEYVVKRTLKEITLKNGEPALSIFDEKTCHLHINPSGKFIIGGPQGDAGLTGRKIIIDTYGGWGAHGGGAFSGKDPTKVDRSAAYITRQMAKSIVTSGLAKRALVQLSYAIGVAKPLSIFVETYGSELGELTADDITDVIKLAYDCRPGAIAKHLQLTEPKYVETAAYCHFGREPRTENGIKYFEWENPVDLSK

>Euglena proxima methionine adenosyltransferase (MATX) mRNA, partial CDS

GAATTCCTCTTCTCTTCAGAATCTGTGAACGAAGGACATCCTGACAAGCTTTGTGATCAGGTCTCTGATTCAGTTTTAGATGCTTGCCTTGCGGTTGATCCAAAAAGTAAGGTGGCCTGCGAAACCGCAGTCAAGGACAACATGGTCATGGTTCTTGGTGAAATTACGACCCAAGCTAAGTTGGACTACGAAACTATTGTTCGGACAGCAGTACGCAACATTGGCTTTGACTCTTTCATAGATGATCTTTCAAGTGTGGACTCTAAGGGCTTGAACTGTGATGATTGTGAGGTCCTGGTGCGCATCAACAAGCAATCTCCTGACATTGCCGGAGGTGTGCACGTTGGCCGCGATGAAATGGACTTTGGGGCTGGCGATCAAGGAATCATGTTTGGGTATGCCAGTGATGAGACCAAGAGTACCATGCCTCTTACCCACTATCTTGCAACACGCCTGGGCAAAACATTGACCGAAGTTCGCAAAGATGGATCTCTATGGTGGCTGCGACCTGATGGGAAGACTCAGGTCACCATCAAGTATAGACAATGTGCAGATGGTTCCGTCGAACCACTCCTCATCCACACTGTCCTGATTTCCACTCAACACGCTGAACCTGGCAAGCGCAAGAAAGGAGAAGAGATCAAAGGGTACAGCGGTCCCGATGCAGATGAATTGGCCCCATCAATGGATAAGATGAATGAGCTCATCATTCAGCACGTCGTAATCCGAACGTTGCAGAATATCACACTGAAGAATGGCGAACCCGCTCTTAGCATCTTCAACCGAGACACATGCCACCTGCACATCAACCCCTCTGGCAAATTCATCATTGGAGGTCCCCAAGGAGACGCTGGGTTGACCGGAAGGAAGATCATCATTGACACATATGGTGGCTGGGGTGCCCACGGAGGTGGTGCCTTTTCTGGCAAGGATCCAACCAAGGTGGATCGATCAGCAGCATACATCACTCGCCAAATTGCCAAGTCCATTGTGACTTCTCAGCTGGCGAAACGTGCCTTGGTGCAATTATCCTATGCCATCGGGGTGGCAAAGCCTCTGTCCATTTTTGTTGAGACCTATGGATCTGAGCAAGGGAGCCTCACCGCCGATGACATCACCGACATCGTAAAATTGGCATTTGATTGCCGTCCAGGTGCCATTGCTAAATCTCTGCAACTGACAGAGCCGAAGTACGTCGAAACCGCAGCTTATTGCCATTTTGGCCGCGAGCCACGCACCGAGAATGGGATCAAGTTCTTTGAGTGGGAAAACCCCGTTGATCTGTCGGCT

>Euglena proxima methionine adenosyltransferase (MATX) amino acid translation, partial protein

EFLFSSESVNEGHPDKLCDQVSDSVLDACLAVDPKSKVACETAVKDNMVMVLGEITTQAKLDYETIVRTAVRNIGFDSFIDDLSSVDSKGLNCDDCEVLVRINKQSPDIAGGVHVGRDEMDFGAGDQGIMFGYASDETKSTMPLTHYLATRLGKTLTEVRKDGSLWWLRPDGKTQVTIKYRQCADGSVEPLLIHTVLISTQHAEPGKRKKGEEIKGYSGPDADELAPSMDKMNELIIQHVVIRTLQNITLKNGEPALSIFNRDTCHLHINPSGKFIIGGPQGDAGLTGRKIIIDTYGGWGAHGGGAFSGKDPTKVDRSAAYITRQIAKSIVTSQLAKRALVQLSYAIGVAKPLSIFVETYGSEQGSLTADDITDIVKLAFDCRPGAIAKSLQLTEPKYVETAAYCHFGREPRTENGIKFFEWENPVDLSA

>Strombomonas accuminata methionine adenosyltransferase (MATX) mRNA, partial CDS

GAGTTCCTTTTTTCCTCTGAATCAGTTAATGAAGGACATCCAGACAAGCTCTGTGACCAAGTGTCAGATTCCGTCCTTGATGCTTGCCTTGCTCAGGACCCGAAGAGCAAAGTGGCTTGTGAGACAGCAGTCAAGGATAATATGGTGATGGTGCTGGGTGAAATTACAACACAGGCAAAATTGGATTATGAGGCCATCGTTCGCAAGGCGGTGCGTAACATTGGTTTCGATTCCTTTGTTGATGACTTGGACAGCGTGGATTCCAAGGGGCTGAATTGTGACGATTGTGAGGTGCTGGTGCGAATCAACAAGCAATCTCCTGATATTGCAGGAGGGGTGCATGTGGGGCGTGATGAAATGGACCTGGGTGCTGGAGACCAGGGCATCATGTTTGGATATGCCAGTGATGAGACGAAGAGCACAATGCCACTGACTCATTACTTGGCAACAAGGTTGGGGAAAACCTTGACGGAGGTGCGGAAGGATGGTACGCTGTGGTGGTTGCGGCCTGATGGCAAGACCCAGGTTACCATCAAGTACAAGCAGTGCGCAGATGGTTCAGTGGAGCCACTGCAAATTCATTCAGTGCTGATCTCCACACAGCACGTGGAACCTGGCAAGCGGAAGAAGAAGGAGGAGATCCGTGGATACCAGGGGGCCGATGGGGATGAAGTGGCACCCACGATGAAGGAAATGAATGAGCACTTGATCGAGCACGTGGTCATCCGCACATTGAAGGGCATCACTCTGAAGAATGGTGAGTCTGCACTCAGCATCTTCGACCGCAACACCTGTCACCTTTACATGAACCCGTCTGGCAAGTTCATCATTGGTGGACCACAAGGCGATGCTGGGCTCACAGGACGCAAAATTATCATCGACACCTACGGTGGTTGGGGAGCTCATGGTGGTGGTGCTTTCTCTGGCAAAGACCCGACCAAGGTGGACAGGTCAGCTGCATACATCACCAGGCAGATTGCCAAGTCGATTGTGGCGTCCAAGTTGGCCAAGCGGGCCCTGGTGCAGCTGTCATATGCCATCGGGGTGGCCAAGCCCCTCTCCATCTTCGTGGAGACATATGGCTCCGAGCAGGGTGCCCTGACTGCCGATGACGTTACGGACATTGTGAAGCTGGCATTCGACTGCCGCCCCGGAGCCATCGCCAAGTCCCTGCAGCTGACTGAGCCCAAGTACACTGAGACGGCCGCATACTGCCACTTTGGCCGTGAGCCCCGCACCGAGAATGGCATCAAGTTCTTCGAGTGGGAGAACCCCGTTGACTTGTCCAAG

>Strombomonas accuminata methionine adenosyltransferase (MATX) amino acid translation, partial protein

EFLFSSESVNEGHPDKLCDQVSDSVLDACLAQDPKSKVACETAVKDNMVMVLGEITTQAKLDYEAIVRKAVRNIGFDSFVDDLDSVDSKGLNCDDCEVLVRINKQSPDIAGGVHVGRDEMDLGAGDQGIMFGYASDETKSTMPLTHYLATRLGKTLTEVRKDGTLWWLRPDGKTQVTIKYKQCADGSVEPLQIHSVLISTQHVEPGKRKKKEEIRGYQGADGDEVAPTMKEMNEHLIEHVVIRTLKGITLKNGESALSIFDRNTCHLYMNPSGKFIIGGPQGDAGLTGRKIIIDTYGGWGAHGGGAFSGKDPTKVDRSAAYITRQIAKSIVASKLAKRALVQLSYAIGVAKPLSIFVETYGSEQGALTADDVTDIVKLAFDCRPGAIAKSLQLTEPKYTETAAYCHFGREPRTENGIKFFEWENPVDLSK

>Petalomonas cantuscygni methionine adenosyltransferase (MAT) mRNA, partial CDS

TTCATCTTCTCGTCCGAGCATGTGACCGAGGGTCACCCGGACAAGATGGCCGACCAGGTGTCGGACCGCATCCTTGATGCGTGCCTCGCCGTCGACCCCCTTGCCAAGGTTGCCTGTGAGTCGGCACTGAAGACCGGTATGGTCATGGTCTTCGGTGAGATCACGACGAAGGCGCACATCGACTACCAGGAGCTCATCCGCGCCACCGTCAAGGACATCGGCTTCGATGACTCGGAGAAGGGGTTCGACTACAAGACGTGCAACGTTCTTGTCGCCATCGAGCAGCAGTCCCCGGAGATCGGCATGTCCGTTCACGGTGATGACGCTAAGTCGCACAGCGATGACCTTGGTGCTGGTGATCAGGGCCTCATGTTCGGCTATGCGACCAACGAGGACGCTGAGACCCTCATGCCGGTTTCCTATGTACTCGCGCGCAACCTCGCGCAGCGCCTCTCCCAGGTGCGCAACGACAAGACCTGCGCGTGGGTGCGCCCGGATGGCAAGACCCAGGTCTCTGTCGAGTACCAGGAGACGAAGGACGCCAACGGCCGGACGAAGCTCACCCCCGTTCGCGTGCACACCATCCTCATCTCCACCCAGCACGCGCCGAGCATCTCCCTGGACGAGATCCGCGCTGAGCTCACGCAGCACGTGATCAAGCCGGTCATCCCGGCCCACCTCCTGGACGACAAGGTCATCCTCCACTTGAACCCCTCCGGGTCCTTCGTCGTCGGTGGCCCCAAGGGCGATGCCGGGCTCACTGGCCGCAAGATCATTGTTGACACCTACGGCGGCTGGGGCGCCCACGGCGGAGGCGCCTTCTCGGGCAAGGACCCCACGAAGGTGGATCGTTCCGCGGCCTACGCGGCCCGCTGGGTCGCGAAGTCGCTCGTGGCCGCGGGCTTCGCGGACCGCGCCCTTGTCCAGGTCGCGTACGCTATTGGGCTGGCGGCCCCCCTCTCGATCCACGTCGAGTCTTATGGCACGGGCAAGGTCCCGGATGCCGTCCTCACGGAGATCGTGAAGCACAACTTCGACCTGCGCCCGCAGCGCATCATTAAGGATCTGGACCTCCGCCGGCCGATCTACTACAAGACGGCGAAGTTCGGCCACTTCGGCCGCACGGACCCCGACTTCACCTGGGAGCAGCCCAAGACCCTCACCCTC

>Petalomonas cantuscygni methionine adenosyltransferase (MAT) amino acid translation, partial protein

FIFSSEHVTEGHPDKMADQVSDRILDACLAVDPLAKVACESALKTGMVMVFGEITTKAHIDYQELIRATVKDIGFDDSEKGFDYKTCNVLVAIEQQSPEIGMSVHGDDAKSHSDDLGAGDQGLMFGYATNEDAETLMPVSYVLARNLAQRLSQVRNDKTCAWVRPDGKTQVSVEYQETKDANGRTKLTPVRVHTILISTQHAPSISLDEIRAELTQHVIKPVIPAHLLDDKVILHLNPSGSFVVGGPKGDAGLTGRKIIVDTYGGWGAHGGGAFSGKDPTKVDRSAAYAARWVAKSLVAAGFADRALVQVAYAIGLAAPLSIHVESYGTGKVPDAVLTEIVKHNFDLRPQRIIKDLDLRRPIYYKTAKFGHFGRTDPDFTWEQPKTLTL

>Phacus orbicularis methionine adenosyltransferase (MAT) mRNA, partial CDS

TCCGAGTCCGTGAACGAGGGCCACCCCGACAAGCTGTGCGACCAGGTGTCTGATGCCGTCCTGGATGCCTGCATCCGCGAGGACCCCACCTCCCGTGTGGCTTGCGAGACCTGCACCAAGACTGGTATGGTGATGATCTTCGGTGAGATCACCACCGCTGCCACCGTCAACTACGAGCAGGTGATCCGCGAGGCTCTGAAGGAGATTGGCTACGATGACGTGAACAAGGGTCTGGACTACAGAACCTGCAACGTGATCGTCGCCATCGAGGAGCAGTCTCCCGATATCGCCCAGTCCGTCGATGCCACCAAGCTGGAGGACATCGGAGCTGGTGACCAGGGCATCATGTTCGGCTACGCCACTGATGAGACCGAGTCTTACATGCCCCTGACCCACAACCTGGCCACCTCTCTGGGCGCCCGCCTGACTGAGGTGCGCAAGACCGGTGTCTGCCCCTGGGTGCGCCCCGACGGCAAGACCCAGGTGACCTGCGAGTACCGCCTGGACAATGGCCGCCCCATCCCCACCCGCGTGCACACCATCGTCATCTCCACCCAGCACTCCGAGGACATCACCCAGGACGAGATCAAGGCCCAGCTGATGGAGCACGTGATCAAGCCCGTGGTGCCCGCCGAGTACCTGGACGAGAAGACCGTGTACCACCTGAACCCCTCTGGCCGCTTCGTCATTGGTGGCCCTCACGGTGATGCCGGTCTGACTGGCCGCAAGATCATCATCGACACCTACGGTGGTTGGGGTGCCCACGGTGGTGGAGCCTTCTCCGGCAAGGACACCACCAAGGTGGACCGCTCCGCTGCCTACGCCGCCCGTTGGGTTGCCAAGTCCCTGGTGGCTGCCGGCCTGGCCCACAGAGTCCTGGTGCAGCTGTCCTACGCCATCGGTGTCTCCTACCCCCTGTCCGTGTTCGTCGACTCCTACGGCACCGGCATCACCCGCTCCGGCAAGACCGATGCCCAGCTGACCGAGATCGTCAAGAACAACTTCGACCTGCGCCCCGGAGGAATCATCCGCGACCTGAACCTGCGCAGACCCGTCCTGAAGAAGACCGCCGCCTACGGCCACTTCGGACGCAACGACCCCGACTTCACCTGGGAGACCCCCAAGCCCCTGACCCTG

>Phacus orbicularis methionine adenosyltransferase (MAT) amino acid translation, partial protein

SESVNEGHPDKLCDQVSDAVLDACIREDPTSRVACETCTKTGMVMIFGEITTAATVNYEQVIREALKEIGYDDVNKGLDYRTCNVIVAIEEQSPDIAQSVDATKLEDIGAGDQGIMFGYATDETESYMPLTHNLATSLGARLTEVRKTGVCPWVRPDGKTQVTCEYRLDNGRPIPTRVHTIVISTQHSEDITQDEIKAQLMEHVIKPVVPAEYLDEKTVYHLNPSGRFVIGGPHGDAGLTGRKIIIDTYGGWGAHGGGAFSGKDTTKVDRSAAYAARWVAKSLVAAGLAHRVLVQLSYAIGVSYPLSVFVDSYGTGITRSGKTDAQLTEIVKNNFDLRPGGIIRDLNLRRPVLKKTAAYGHFGRNDPDFTWETPKPLTL

**Species tree topologies used for topology tests**

MATX data set

SSU rRNA tree

(Thalassionema_nitzschioides,Asterionella_glacialis,(Cylindrotheca_closterium,((Ditylum_brightwellii,(Thalassiosira_pseudonana,(Detonula_confervacea,Skeletonema_costatum))),(Aureococcus_anophagefferens,((Heterocapsa_triquetra,(Karlodinium_veneficum,(Karenia_brevis,(Amphidinium_carterae,(Lingulodinium_polyedrum,Alexandrium_catenella))))),((Lactuca_serriola,((((Phacus_inflexus,Phacus_orbicularis),(Lepocinclis_playfairiana,Lepocinclis_tripteris)),(Euglena_proxima,(((Euglena_viridis,Euglena_stellata),(Euglena_clara,(Euglena_gracilis,Euglena_hiemalis))),((Euglenaria_anabena,(Monomorphina_aenigmatica,(Monomorphina_parapyrum,Monomorphina_pyrum))),((Trachelomonas_sp,Trachelomonas_volvocina),(Strombomonas_accuminata,Trachelomonas_ellipsoidalis)))))),(Eutreptia_viridis,(Eutreptiella_gymnastica,Eutreptiella_braarudii)))),(Dendroctonus_frontalis,(Prymnesium_parvum,(Emiliania_huxleyi,Isochrysis_galbana)))))))));

Manually constructed tree

(Thalassionema_nitzschioides,Asterionella_glacialis,(Cylindrotheca_closterium,((Ditylum_brightwellii,(Thalassiosira_pseudonana,(Detonula_confervacea,Skeletonema_costatum))),(Aureococcus_anophagefferens,((Heterocapsa_triquetra,(Karlodinium_veneficum,(Karenia_brevis,(Amphidinium_carterae,(Lingulodinium_polyedrum,Alexandrium_catenella))))),((Lactuca_serriola,(Dendroctonus_frontalis,((((Phacus_inflexus,Phacus_orbicularis),(Lepocinclis_playfairiana,Lepocinclis_tripteris)),(Euglena_proxima,(((Euglena_viridis,Euglena_stellata),(Euglena_clara,(Euglena_gracilis,Euglena_hiemalis))),((Euglenaria_anabena,(Monomorphina_aenigmatica,(Monomorphina_parapyrum,Monomorphina_pyrum))),(((Trachelomonas_sp,Trachelomonas_volvocina),Trachelomonas_ellipsoidalis),Strombomonas_accuminata))))),(Eutreptia_viridis,(Eutreptiella_gymnastica,Eutreptiella_braarudii))))),(Prymnesium_parvum,(Emiliania_huxleyi,Isochrysis_galbana))))))));

MATX data set excluding *Aureococcus_anophagefferenss*, *Prymnesium*, *Lactuca* and *Dendroctonus*

SSU rRNA tree

(Thalassionema_nitzschioides,Asterionella_glacialis,(Cylindrotheca_closterium,(((Heterocapsa_triquetra,(Karlodinium_veneficum,(Karenia_brevis,(Amphidinium_carterae,(Lingulodinium_polyedrum,Alexandrium_catenella))))),(((((Phacus_inflexus,Phacus_orbicularis),(Lepocinclis_tripteris,Lepocinclis_playfairiana)),(Euglena_proxima,(((Strombomonas_accuminata,(Trachelomonas_ellipsoidalis,(Trachelomonas_volvocina,Trachelomonas_sp))),(Euglenaria_anabena,(Monomorphina_aenigmatica,(Monomorphina_pyrum,Monomorphina_parapyrum)))),(Euglena_clara,((Euglena_gracilis,Euglena_hiemalis),(Euglena_stellata,Euglena_viridis)))))),(Eutreptia_viridis,(Eutreptiella_gymnastica,Eutreptiella_braarudii))),(Isochrysis_galbana,Emiliania_huxleyi))),(Ditylum_brightwellii,(Thalassiosira_pseudonana,(Detonula_confervacea,Skeletonema_costatum))))));

Manually constructed tree

(Thalassionema_nitzschioides,Asterionella_glacialis,(Cylindrotheca_closterium,(((Heterocapsa_triquetra,(Karlodinium_veneficum,(Karenia_brevis,(Amphidinium_carterae,(Lingulodinium_polyedrum,Alexandrium_catenella))))),(((((Phacus_inflexus,Phacus_orbicularis),(Lepocinclis_tripteris,Lepocinclis_playfairiana)),(Euglena_proxima,(((Strombomonas_accuminata,(Trachelomonas_ellipsoidalis,(Trachelomonas_volvocina,Trachelomonas_sp))),(Euglenaria_anabena,(Monomorphina_aenigmatica,(Monomorphina_pyrum,Monomorphina_parapyrum)))),((Euglena_clara,(Euglena_gracilis,Euglena_hiemalis)),(Euglena_stellata,Euglena_viridis))))),(Eutreptia_viridis,(Eutreptiella_gymnastica,Eutreptiella_braarudii))),(Isochrysis_galbana,Emiliania_huxleyi))),(Ditylum_brightwellii,(Thalassiosira_pseudonana,(Detonula_confervacea,Skeletonema_costatum))))));

Rooted MATX data set

SSU rRNA tree

(Thalassionema_nitzschioides,Asterionella_glacialis,(Cylindrotheca_closterium,((Aureococcus_anophagefferens,(((Heterocapsa_triquetra,(Karlodinium_veneficum,(Karenia_brevis,(Amphidinium_carterae,(Lingulodinium_polyedrum,Alexandrium_catenella))))),(Dendroctonus_frontalis,Lactuca_serriola)),((Prymnesium_parvum,(Isochrysis_galbana,Emiliania_huxleyi)),((TricVagi,EscherichK),((Euglena_proxima,(((Lepocinclis_playfairiana,Lepocinclis_tripteris),(Phacus_orbicularis,Phacus_inflexus)),(((Trachelomonas_ellipsoidalis,Strombomonas_accuminata),(Trachelomonas_sp,Trachelomonas_volvocina)),(((Euglena_stellata,Euglena_viridis),(Euglena_clara,(Euglena_gracilis,Euglena_hiemalis))),(Euglenaria_anabena,(Monomorphina_aenigmatica,(Monomorphina_parapyrum,Monomorphina_pyrum))))))),(Eutreptia_viridis,(Eutreptiella_braarudii,Eutreptiella_gymnastica))))))),(Ditylum_brightwellii,(Thalassiosira_pseudonanai,(Detonula_confervacea,Skeletonema_costatum))))));

Manually constructed tree

((TricVagi,EscherichK),(((((Lepocinclis_playfairiana,Lepocinclis_tripteris),(Phacus_orbicularis,Phacus_inflexus)),(Euglena_proxima,((Strombomonas_accuminata,(Trachelomonas_ellipsoidalis,(Trachelomonas_sp,Trachelomonas_volvocina))),(((Euglena_stellata,Euglena_viridis),(Euglena_clara,(Euglena_gracilis,Euglena_hiemalis))),(Euglenaria_anabena,(Monomorphina_aenigmatica,(Monomorphina_parapyrum,Monomorphina_pyrum))))))),(Eutreptia_viridis,(Eutreptiella_braarudii,Eutreptiella_gymnastica))),Dendroctonus_frontalis,(Lactuca_serriola,((Prymnesium_parvum,(Isochrysis_galbana,Emiliania_huxleyi)),((Heterocapsa_triquetra,(Karlodinium_veneficum,(Karenia_brevis,(Amphidinium_carterae,(Lingulodinium_polyedrum,Alexandrium_catenella))))),(Aureococcus_anophagefferens,((Ditylum_brightwellii,(Thalassiosira_pseudonana,(Detonula_confervacea,Skeletonema_costatum))),(Cylindrotheca_closterium,(Asterionella_glacialis,Thalassionema_nitzschioides)))))))));

Rooted MATX data set excluding *Aureococcus_anophagefferenss*, *Prymnesium*, *Lactuca* and *Dendroctonus*

SSU rRNA tree

(Thalassionema_nitzschioides,Asterionella_glacialis,(Cylindrotheca_closterium,(((Heterocapsa_triquetra,(Karlodinium_veneficum,(Karenia_brevis,(Amphidinium_carterae,(Alexandrium_catenella,Lingulodinium_polyedrum))))),((Isochrysis_galbana,Emiliania_huxleyi),((TricVagi,EscherichK),((Euglena_proxima,(((Phacus_inflexus,Phacus_orbicularis),(Lepocinclis_tripteris,Lepocinclis_playfairiana)),((((Euglena_stellata,Euglena_viridis),(Euglena_clara,(Euglena_gracilis,Euglena_hiemalis))),(Euglenaria_anabena,(Monomorphina_aenigmatica,(Monomorphina_parapyrum,Monomorphina_pyrum)))),((Strombomonas_accuminata,Trachelomonas_ellipsoidalis),(Trachelomonas_volvocina,Trachelomonas_sp))))),(Eutreptia_viridis,(Eutreptiella_gymnastica,Eutreptiella_braarudii)))))),(Ditylum_brightwellii,(Thalassiosira_pseudonanai,(Skeletonema_costatum,Detonula_confervacea))))));

Manually constructed tree

((TricVagi,EscherichK),(((((Lepocinclis_playfairiana,Lepocinclis_tripteris),(Phacus_orbicularis,Phacus_inflexus)),(Euglena_proxima,((Strombomonas_accuminata,(Trachelomonas_ellipsoidalis,(Trachelomonas_sp,Trachelomonas_volvocina))),(((Euglena_stellata,Euglena_viridis),(Euglena_clara,(Euglena_gracilis,Euglena_hiemalis))),(Euglenaria_anabena,(Monomorphina_aenigmatica,(Monomorphina_parapyrum,Monomorphina_pyrum))))))),(Eutreptia_viridis,(Eutreptiella_braarudii,Eutreptiella_gymnastica))),((Isochrysis_galbana,Emiliania_huxleyi),((Heterocapsa_triquetra,(Karlodinium_veneficum,(Karenia_brevis,(Amphidinium_carterae,(Lingulodinium_polyedrum,Alexandrium_catenella))))),((Ditylum_brightwellii,(Thalassiosira_pseudonana,(Detonula_confervacea,Skeletonema_costatum))),(Cylindrotheca_closterium,(Asterionella_glacialis,Thalassionema_nitzschioides)))))));

MATX data set of euglenids

SSU rRNA tree

(Monomorphina_pyrum,Monomorphina_parapyrum,(Monomorphina_aenigmatica,(Euglenaria_anabena,((Strombomonas_accuminata,(Trachelomonas_ellipsoidalis,(Trachelomonas_sp,Trachelomonas_volvocina))),(((Euglena_stellata,Euglena_viridis),(Euglena_clara,(Euglena_gracilis,Euglena_hiemalis))),(((Lepocinclis_playfairiana,Lepocinclis_tripteris),(Phacus_inflexus,Phacus_orbicularis)),(Euglena_proxima,(Eutreptia_viridis,(Eutreptiella_braarudii,Eutreptiella_gymnastica)))))))));

Manually constructed tree

(Monomorphina_pyrum,Monomorphina_parapyrum,(Monomorphina_aenigmatica,(Euglenaria_anabena,((Strombomonas_accuminata,(Trachelomonas_ellipsoidalis,(Trachelomonas_sp,Trachelomonas_volvocina))),(((Euglena_stellata,Euglena_viridis),(Euglena_clara,(Euglena_gracilis,Euglena_hiemalis))),(Euglena_proxima,(((Lepocinclis_playfairiana,Lepocinclis_tripteris),(Phacus_inflexus,Phacus_orbicularis)),(Eutreptia_viridis,(Eutreptiella_braarudii,Eutreptiella_gymnastica)))))))));
